# Tunable mechanical, corrosion, and antimicrobial performance in multifunctional Cu_x_‑Cr-Fe-Mn-Ni high-entropy alloys and E-beam coatings

**DOI:** 10.1038/s41598-026-55056-0

**Published:** 2026-06-14

**Authors:** Bogdan Postolnyi, Dumitru Mitrica, Arcadii Sobetkii, Aizhan B. Talipova, Krzysztof Rokosz, Volodymyr Buranych, Laurentiu-Florin Mosinoiu, Martin Kusý, Feodor M. Borodich, Vladimir A. Levchenko, Laura-Madalina Cursaru, Alexander Pogrebnjak

**Affiliations:** 1 Institute of Physics for Advanced Materials, Nanotechnology and Photonics, Faculty of Sciences, University of Porto, Porto, 4169-007 Portugal; 2 National R&D Institute for Non−Ferrous and Rare Metals, Pantelimon, 077145 Romania; 3 Sumy State University, 116 Kharkivska st, Sumy, 40007 Ukraine; 4 Department of Biotechnology, Al-Farabi Kazakh National University, Almaty, 050040 Kazakhstan; 5 Division of Surface Electrochemistry & Technology, Faculty of Mechanical Engineering, Koszalin University of Technology, Racławicka 15-17, Koszalin, 75-620 PL Poland; 6 Institute of Electrical Engineering, Slovak Academy of Sciences, Dubravska cesta 9, Bratislava, 84104 Slovakia; 7 Institute of Materials, Faculty of Materials Science and Technology in Trnava, Slovak University of Technology in Bratislava, Trnava, 917 24 Slovakia; 8 College of Aerospace Engineering, Chongqing University, Chongqing, 400030 China; 9 Institute of Advanced Coatings and Materials, Taizhou University, Taizhou, 318000 China

**Keywords:** Multicomponent alloys, Functional surface engineering, Protective coatings, Corrosion resistance, Antimicrobial surfaces, Microbiologically influenced corrosion, Chemistry, Engineering, Materials science

## Abstract

Cu_x_-Cr-Fe-Mn-Ni high-entropy alloys and their electron-beam-deposited coatings demonstrate a tunable balance of mechanical strength, corrosion resistance, and antimicrobial performance, offering a new paradigm for multifunctional protective materials. Dense nanocrystalline coatings with complex phase coexistence achieved exceptional hardness (~ 9.4 GPa) and a high elastic strain-to-failure ratio (H/E ≈ 0.05), indicating potential for improved resistance to wear and contact damage. Controlled Cu content governs surface oxide composition, simultaneously enhancing corrosion protection and enabling targeted bactericidal activity against *Pseudomonas aeruginosa* (up to 71% reduction), mitigating microbiologically influenced corrosion (MIC) in saline environments. These results reveal a critical interplay between phase structure, surface chemistry, and functional performance, establishing a rational pathway for designing high-performance coatings and bulk alloys with environment-specific multifunctionality. Cu_x_-Cr-Fe-Mn-Ni HEAs are versatile candidates for industrial, marine, and energy applications where tailored mechanical, chemical, and antimicrobial properties are required.

**Supplementary Information:**

The online version contains supplementary material available at 10.1038/s41598-026-55056-0.

## Introduction

High-entropy materials and, in particular, high-entropy alloys (HEAs), with over 20 years of formal history and development^1,2^, emerged as a well-recognized class of advanced materials with remarkable potential across diverse applications. HEAs are typically composed of five or more principal metallic elements (each within 5–35 at%). Their distinctive core effects, such as high configurational entropy, sluggish diffusion, lattice distortion, and cocktail effect, contribute to a unique combination of superior mechanical properties, toughness, and enhanced thermal and corrosion resistance, as extensively documented and studied in the literature^3–8^.

The vast compositional space of HEAs offers nearly infinite combinations of elements, often resulting in unexpected synergistic property enhancements. This diversity continues to drive research, promising decades of further investigation and discovery. Emerging technologies such as machine learning (ML)^9–11^ and artificial intelligence (AI)^12,13^ are poised to accelerate HEA development by enabling faster materials screening, prediction of desired properties, and potentially closed-loop, autonomous materials design through materials acceleration platforms (MAPs)^14^. Nonetheless, the foundation of these advances remains rooted in experimental materials science, as computational predictions and ML models require experimental validation and high-quality datasets.

In recent years, research on HEAs has progressed from fundamental studies of intrinsic properties to functionalization for real-world industrial applications. Of particular interest are environments requiring long-term stability under extreme conditions, such as high-performance machining, aerospace, and radiation-resistant applications. More recently, HEAs have also attracted attention for mitigating microbiologically influenced corrosion (MIC) through antimicrobial functionalization using intrinsically antimicrobial metallic elements^8,15,16^. Achieving an optimal combination of wear and corrosion resistance with antimicrobial activity is crucial for enabling dual-action surface protection in corrosive and microbiologically active environments, including water treatment, energy, and marine industries.

Building upon the design principles of the well-known Cantor-type CoCrFeMnNi alloy^2^, which has become a widely studied benchmark system focused primarily on mechanical properties and fundamental concepts, the Cu-Cr-Fe-Mn-Ni system represents a compositional and functional evolution toward multifunctional, application-oriented high-entropy materials. In this context, the incorporation of Cu instead of Co introduces further structural and chemical variation, providing opportunities to tailor mechanical behavior, corrosion resistance, and antimicrobial functionality. Considering the strong influence of both composition and fabrication route on phase formation and performance, systematic studies of such Cu-containing derivatives are essential. The Cu-Cr-Fe-Mn-Ni alloy family therefore presents a promising platform for exploring the interplay between structural effects, corrosion protection, and antimicrobial response, enabling multifunctional performance, including its potential for MIC mitigation.

In this work, Cu-Cr-Fe-Mn-Ni HEAs were investigated both as bulk materials and as E-beam deposited coatings. Comprehensive characterization was carried out to assess the structural and phase composition, mechanical properties, corrosion behavior, and antimicrobial performance — key factors determining their potential for long-term surface protection in corrosive and microbiologically active environments.

## Experimental details

### Alloy fabrication

The Cu_x_-Cr-Fe-Mn-Ni HEAs with Cu 5 and 10% fraction were synthesized from pure elemental mixtures. Alloy fabrication was carried out in a vacuum induction melting furnace (Model MFG 30, Linn High Therm GmbH). A calcium oxide (CaO) ceramic crucible was used during melting and alloy homogenization to minimize contamination. Each alloy charge, approximately 700 g, was melted and subsequently cast into copper molds under a controlled argon atmosphere to prevent oxidation. The alloys were prepared with the following nominal compositions: 5%CuHEA (Cu 5 at%, Cr 23 at%, Fe 24 at%, Mn 24 at%, Ni 25 at%) and 10%CuHEA (Cu 10 at%, Cr 22 at%, Fe 23 at%, Mn 22 at%, Ni 23 at%). The primary compositional difference between the two alloys was the Cu content. High-purity elemental metals (Cu, Cr, Fe, Mn, and Ni; ≥99.9 wt% purity, obtained from certified commercial suppliers) were used as starting materials to ensure consistent alloy quality across all compositions. The comprehensive characterization of the bulk HEAs was conducted not only to provide target materials for coating deposition, but also to establish intrinsic structure–property relationships and assess their potential applicability as standalone structural and functional materials.

### Coatings deposition

The ingots produced from the Cu_x_-Cr-Fe-Mn-Ni HEAs were used as source materials for electron beam (E-beam) physical vapor deposition. Coatings were deposited under high-vacuum conditions onto stainless steel 304 L substrates (15 × 15 × 3 mm). Silicon substrates were additionally used for selected high-resolution imaging and elemental analyses. The substrate temperature was maintained either at room temperature (RT) or at 400 °C. The films deposited at room temperature exhibited poor adhesion and partial delamination, likely due to residual thermal stresses and insufficient interfacial bonding. In contrast, coatings deposited at a substrate temperature of 400 °C showed uniform morphology, strong adhesion, and structural integrity. Accordingly, only coatings deposited at 400 °C were selected for further characterization. The choice of a substrate temperature of 400 °C is consistent with previous PVD studies on HEA thin films, which report amorphous growth at RT and crystallization at elevated substrate temperatures around 400 °C^17^.

### Elemental and morphological analysis

The chemical composition of the Cu_x_-Cr-Fe-Mn-Ni alloy samples was determined by inductively coupled plasma optical emission spectroscopy (ICP-OES) using an Agilent 725 spectrometer (Agilent Technologies, Santa Clara, CA, USA). Optical microscopy was performed using a Carl Zeiss optical microscope to evaluate the general morphology of the as-cast alloys.

For microstructural analysis, metallographic specimens were prepared by standard grinding and polishing procedures using grinding papers of various roughness, followed by final polishing with a diamond suspension. To reveal the solidification microstructure for scanning electron microscopy (SEM) observation, the polished surfaces were chemically etched using a standard aqua regia–type solution, followed by rinsing and drying.

Detailed microstructural and compositional analyses were conducted using a FEI Quanta 250 scanning electron microscope (FEI Company, Eindhoven, The Netherlands) equipped with an EDAX energy-dispersive X-ray spectroscopy (EDS) system (EDAX, USA). EDS was used to provide semi-quantitative compositional information, primarily to confirm elemental presence and assess relative compositional uniformity rather than precise quantitative composition. Analyses were performed at an accelerating voltage of 15 kV using an ELEMENT Silicon Drift Detector (SDD) and the Element EDS Analysis Software Suite to determine local chemical compositions (at%). Surface feature (particle) size distribution was quantified from SEM micrographs using automated image analysis. Feature size was defined as the equivalent circular diameter derived from the projected area of segmented surface features. Image segmentation was performed using contrast enhancement, thresholding, and morphological filtering. Large surface agglomerates associated with the deposition process were excluded to focus on the characteristic coating morphology.

### Structural analysis

Phase identification and structural characterization were performed using a Bruker D8 ADVANCE powder X-ray diffractometer (Bruker AXS GmbH, Karlsruhe, Germany) equipped with a Cu Kα radiation source and a scintillation detector operating in θ–θ geometry. Diffraction data were collected in the 2θ range of 20°–100° with a step size of 0.02°. For coatings, X-ray diffraction (XRD) analysis was conducted in grazing incidence mode (incident angle 1°) to minimize substrate signal contribution. Data acquisition was performed using DIFFRACplus XRD Commander (Bruker AXS), and phase analysis was carried out using DIFFRAC.EVA v5.1 (2019) and Crystal Impact Match! software with reference data from the Crystallography Open Database (COD 2025; entries 9012713, 4113931, 1512502, 9008589, corresponding to FCC, BCC, and α-Mn–type structures). Quantitative phase fractions for bulk alloys were estimated using the Reference Intensity Ratio (RIR) method^18,19^.

### Mechanical characterization

Vickers microhardness measurements of the bulk HEA alloys were performed using a Wilson VH3100 hardness tester (Wilson Instruments) equipped with a single indenter system and a load cell range of 0.05–10 kgf, as described in detail in our previous work^20^. The reported values correspond to the average of multiple indentations.

Nanohardness and reduced Young’s modulus were evaluated using an Anton Paar NHT nanoindenter (Anton Paar GmbH, Graz, Austria) equipped with a Berkovich tip at a maximum load of 5.00 mN. The dwell time (pause) at maximum load was set to 5.0 s, while both the loading and unloading rates were 15.00 mN/min. Data were acquired at an Advanced Acquisition Rate of 10.0 Hz. The load-displacement curves were processed by the Oliver and Pharr method^21^ to calculate the final properties. In order to avoid the substrate contribution, indentation depths did not exceed 10% of the overall thickness. The H/E ratio, also known as the elastic strain to failure index, was then calculated to evaluate the material’s inherent resistance to plastic deformation.

### Electrochemical corrosion tests

Corrosion behavior of CuCrFeMnNi HEA, in bulk form and as coatings on 304 L stainless steel (SS), was evaluated alongside uncoated SS. Tests were performed at room temperature in a 1 L ASTM corrosion cell with a 1 cm² exposed working electrode. A three-electrode configuration was used: Ag/AgCl reference electrode, platinum counter-electrode, and the sample as the working electrode, connected to an Autolab PGSTAT 128 N potentiostat with NOVA 2.1 software. The electrolyte was 3.5 wt% NaCl (35 g/L), in accordance with ASTM G61-86 and commonly used to simulate marine saline environments. After 60 min of immersion, linear polarization measurements were conducted by scanning ± 0.1 V around the open-circuit potential at 0.001 V/s.

Corrosion parameters including observed corrosion potential *E*_corr_, corrosion current density *j*_corr_, corrosion current *i*_corr,_ corrosion rate, and polarization resistance *R*_p_ were derived from the Tafel analysis. The corrosion current density *j*_corr_ was determined by Tafel extrapolation of the linear polarization curves, and the corrosion rate was calculated from *j*_corr_ according to Faraday’s law using the alloy equivalent weight and density. *R*_p_ was obtained from the slope of the polarization curve in the vicinity of *E*_corr_.

### Antimicrobial benchmark details

To evaluate the antibacterial potential of the HEA materials, antimicrobial activity was assessed using complementary approaches applied to different material forms. The agar diffusion and time–kill assays were performed on bulk HEA samples, whereas the long-term MIC experiments were conducted on HEA coatings deposited on stainless steel substrates.

#### Agar diffusion method

Mueller–Hinton agar medium was sterilized by autoclaving and poured into sterile Petri dishes to form a uniform 4 mm-thick layer. The plates were allowed to solidify at room temperature. Suspensions of the test microorganisms, *Staphylococcus aureus* ATCC 6538 and *Pseudomonas aeruginosa* ATCC 9027, were prepared from pure 24-hour cultures grown on a solid nutrient medium. *P. aeruginosa* was selected due to its relevance in MIC in marine and water environments, while *S. aureus* was included as a representative Gram-positive reference strain to evaluate potential differences in antimicrobial response. Several uniform, well-isolated colonies were selected and transferred with a sterile loop into tubes containing sterile physiological saline. The turbidity of the suspension was adjusted to 0.5 on the McFarland standard, corresponding to approximately 1.5 × 10⁸ CFU/mL.

Two milliliters of the inoculum were pipetted onto the surface of the agar and evenly distributed by gentle tilting. Excess liquid was removed, and the plates were air-dried for 10 min at room temperature. The alloy samples were then placed on the agar surface using sterile forceps. The prepared plates were incubated at 35 °C for 24 h. After incubation, the diameters of the bacterial growth inhibition zones were measured with an accuracy of 1 mm. The antimicrobial activity was classified as follows: no activity (≤ 5 mm), low (5–10 mm), moderate (10–17 mm), and high (≥ 17 mm).

#### Time-kill assay for bulk HEAs

The time–kill test was performed according to the CLSI M26-A guidelines. One milliliter of each alloy sample was added to test tubes containing liquid nutrient medium inoculated with the target microorganisms at an initial concentration of 5 × 10⁵ CFU/mL. The tubes were incubated in a shaker at 37 °C for 24 h. A control tube containing the same medium and bacterial inoculum but without the sample served as the reference.

At specific time intervals (0, 1, 6, 10, and 24 h), 100 µL of bacterial suspension was collected from each tube and plated on selective agar media: nutrient salt agar (NSA) for *S. aureus* and Pseudomonas selective agar (PSA) for *P. aeruginosa*. The plates were incubated at 37 °C for 48 h, and the number of colony-forming units (CFU/mL) was determined to estimate the number of viable cells.

The reduction in bacterial count *R* was calculated using the following equation: *R* (%) = [(*A*-*B)*/*A*]×100%: where *A* is the bacterial count in the control group (without the sample) and *B* is the bacterial count in the test group (with the sample). In general, according to this approach, a material is considered to exhibit a high-level bactericidal effect if it causes at least 90% bacterial cell death within 6 h, corresponding to an estimated 99.9% of reduction in bacterial viability after 24 h; lower levels of cell death were also considered indicative of antimicrobial activity.

#### Long-term antimicrobial evaluation of HEA coatings

In addition to the bulk HEA samples, further testing was performed on 304 L stainless steel coupons coated with Cu_x_-Cr-Fe-Mn-Ni (5% and 10% Cu) to evaluate their resistance to microbial corrosion induced by *P. aeruginosa*. The long-term experiments were conducted on coated samples because the coatings represent the functional surface intended for practical applications, particularly in marine and saline environments where MIC is relevant. Coupons were sterilized by ethanol immersion and UV exposure prior to immersion in 3.5 wt% NaCl saline solution inoculated with a consortium of three *P. aeruginosa* strains. *P. aeruginosa* was selected as a model MIC-related organism due to its well-documented ability to form biofilms and induce corrosion in marine and industrial water systems. Abiotic controls were prepared in parallel. The biotic and abiotic samples were incubated at 30 °C for 60 days. Bacterial growth in the saline solution was monitored by optical density at 660 nm (OD_660_) and by plating on selective media to assess cell viability, pigment production, and biofilm formation. At the end of the incubation period, coupons were analyzed for macroscopic and microscopic surface changes, and the pH of the solution was measured.

Coupons were incubated in 3.5 wt% NaCl solution for 60 days to allow for the development of mature biofilms and to simulate long-term microbial exposure, which is relevant for assessing microbiologically influenced corrosion (MIC) under near-realistic conditions.

#### Surface chemistry analysis by X-ray photoelectron spectroscopy

X-ray photoelectron spectroscopy (XPS) measurements of CuCrFeMnNi HEA coatings were performed using a SCIENCE SES 2002 instrument equipped with a monochromatic Al Kα X-ray source (hν = 1486.6 eV, 18.7 mA, 13.02 kV). Spectra were acquired from an analysis area of 1 × 3 mm with a pass energy of 500 eV, energy step of 0.2 eV, and step time of 200 ms. The spectrometer binding energy was calibrated using the Fermi level of a clean metallic sample. Experiments were conducted under ultra-high vacuum (~ 6 × 10⁻⁸ Pa) and normal emission geometry.

XPS spectra were analyzed using CasaXPS 2.3.14 software with Shirley background subtraction. The binding energy scale was referenced to the Fermi level. The accuracy of the energy scale was confirmed by the position of the metallic Cu 2p_3/2_ peak at 932.6 eV^22^ observed after sputtering, with no additional charge correction applied. Both survey and high-resolution scans were obtained to determine surface composition, oxidation states, and the effects of sputtering on the native oxide layer. The residual oxide thickness after sputtering was estimated from the attenuation of the photoelectron signal from the underlying metallic HEA, using the oxide/metal peak-area ratio in the high-resolution spectra and standard inelastic mean free path values for photoelectrons^23^.

## Results and discussion

### HEA chemical composition of bulk samples

The bulk chemical compositions of the Cu_x_-Cr-Fe-Mn-Ni HEAs, determined by ICP-OES, are summarized in Table 1. The measured values confirm that the alloys closely match the nominal compositions, with minor deviations likely arising from element volatilization during vacuum induction melting.

**Table 1:** Bulk chemical composition of Cuₓ–Cr–Fe–Mn–Ni alloys (at%).

Name	Cu		Cr		Fe		Mn		Ni	
	wt%	at%	wt%	at%	wt%	at%	wt%	at%	wt%	at%
Sample 1	5.9	5.3	20.9	22.6	23.8	24.1	23.1	23.8	26.3	25.3
Sample 2	11.5	10.2	20.6	22.3	22.9	23.1	19.8	20.3	25.2	24.2

The data indicate a slight increase in Cu content from Sample 1 to Sample 2, accompanied by a small decrease in Mn, while the concentrations of Cr, Fe, and Ni remain relatively stable. These variations are consistent with targeted alloy design and are expected to influence both the microstructural evolution and functional properties of the alloys.

The Cu contents of 5 and 10 at% were selected to represent moderate and elevated Cu additions within the Cu–Cr–Fe–Mn–Ni system, enabling assessment of compositional effects while preserving the high-entropy alloy framework. Similar Cu levels have been explored in Cu-containing derivatives of Cantor-type HEAs, where Cu is known to affect phase stability, elemental partitioning, and functional properties without fundamentally altering the base alloy system^24,25^.

### Microstructural observations and mechanical properties of the alloys

**Fig. 1:**
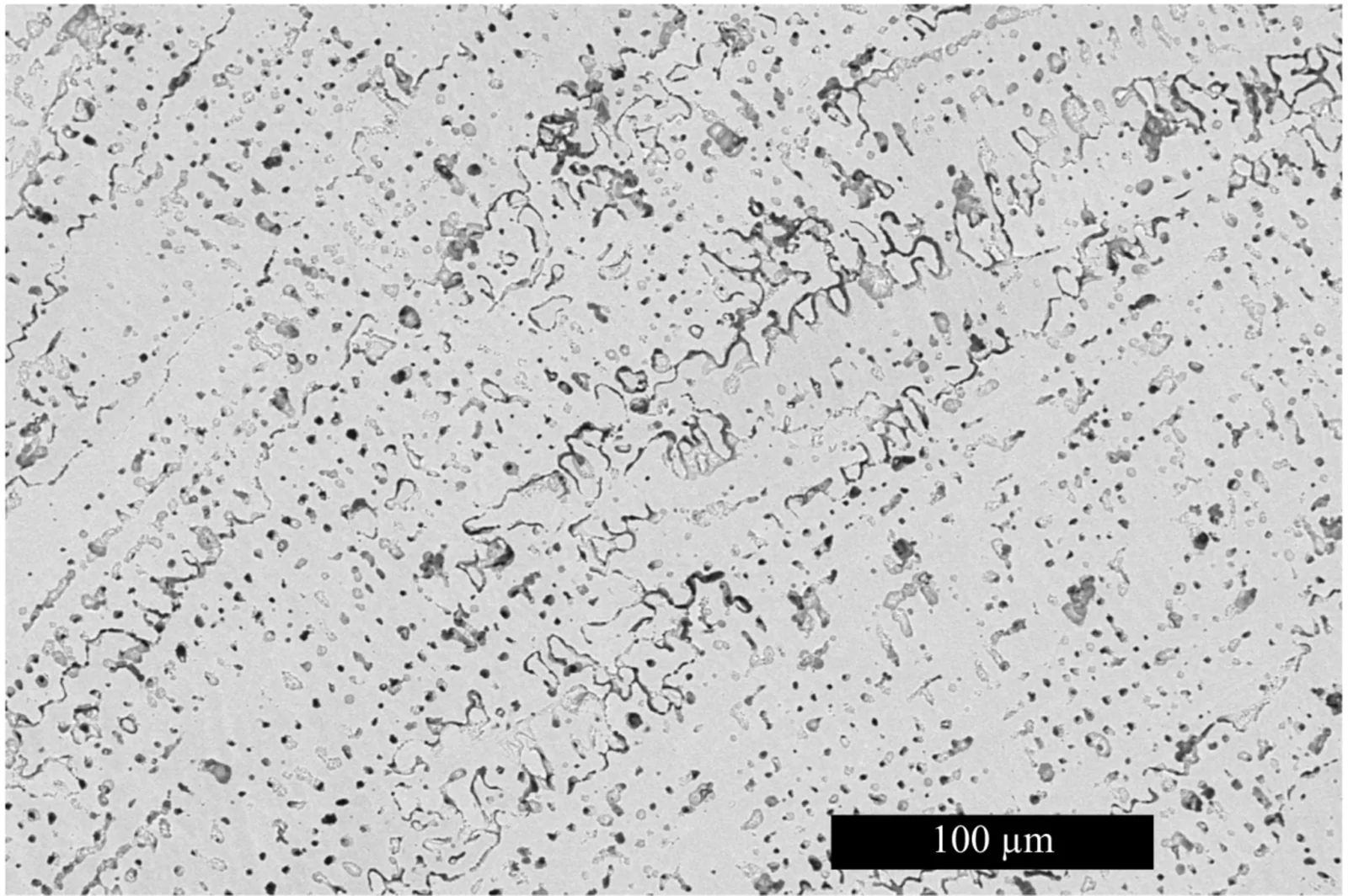
SEM image of the surface of 10%CuHEA showing a pronounced dendritic microstructure with large dendrite arms.

Figure 1 presents an SEM image of the etched surface of the 10%CuHEA alloy. The microstructure is characterized by a predominantly dendritic morphology, with small interdendritic regions of comparable contrast. This dendritic structure reflects the solidification behavior during casting, where the primary dendrites solidify first, followed by solute redistribution in the interdendritic areas. The interdendritic regions appear evenly distributed and exhibit comparable contrast across the observed area, indicating morphological uniformity at the examined length scale. At the examined length scale, both investigated alloys exhibit comparable dendritic features.

**Fig. 2:**
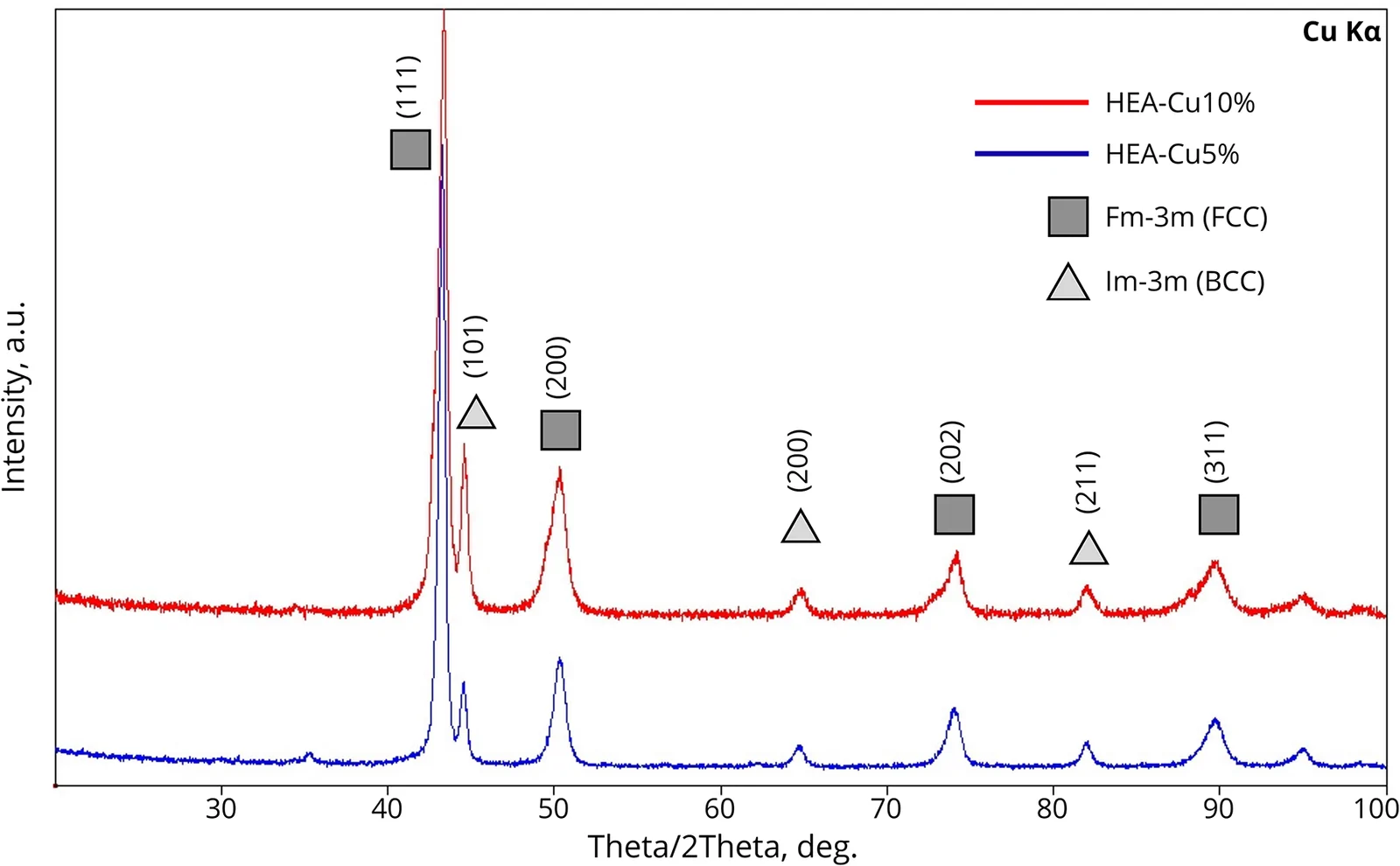
XRD patterns of 5%CuHEA and 10%CuHEA measured using Cu K*α* radiation, showing dominant FCC phase with a minor BCC contribution.

XRD analysis of the as-cast alloys (Fig. 2) reveals a dominant face-centered cubic (FCC) phase (space group Fm-3 m, 225) along with minor contributions from a body-centered cubic (BCC) phase (space group Im-3 m, 229). In both 5%CuHEA and 10%CuHEA alloys, the FCC phase is predominant; however, the intensity of the BCC peaks is slightly higher in the 10%CuHEA alloy, suggesting a larger BCC fraction. Quantitative analysis indicates that the BCC phase fraction increases from approximately 10% in 5%CuHEA to 15% in 10%CuHEA, highlighting that increasing Cu content favors either the formation or detectability of the BCC phase. These results demonstrate that while FCC remains the main crystal structure in both alloys, subtle changes in composition can influence the presence of secondary BCC phases, which may impact mechanical properties.

Correspondingly, the mechanical behavior of the alloys further supports the structural observations. The microhardness measurements show an increase from 240 HV for the 5%CuHEA to 290 HV for the 10%CuHEA alloy, a notable rise of approximately 21%. This enhancement can be attributed to the combined influence of the slightly higher BCC phase fraction and the more pronounced dendritic morphology observed in the 10%CuHEA sample, compared to the less defined structure of 5%CuHEA as studied in our previous work^20^. The BCC structure generally contributes higher hardness compared to the FCC phase, as BCC lattices exhibit higher intrinsic resistance to dislocation motion and typically provide greater strength in multiphase HEA systems^26–28^. In addition, the presence of well-developed dendritic regions and Cu-induced microsegregation may further strengthen the material by impeding dislocation motion. These microstructural and phase-related effects act synergistically, resulting in the higher hardness of the 10%CuHEA alloy.

### Elemental composition and morphology of Cu_x_-Cr-Fe-Mn-Ni HEA coatings

**Fig. 3:**
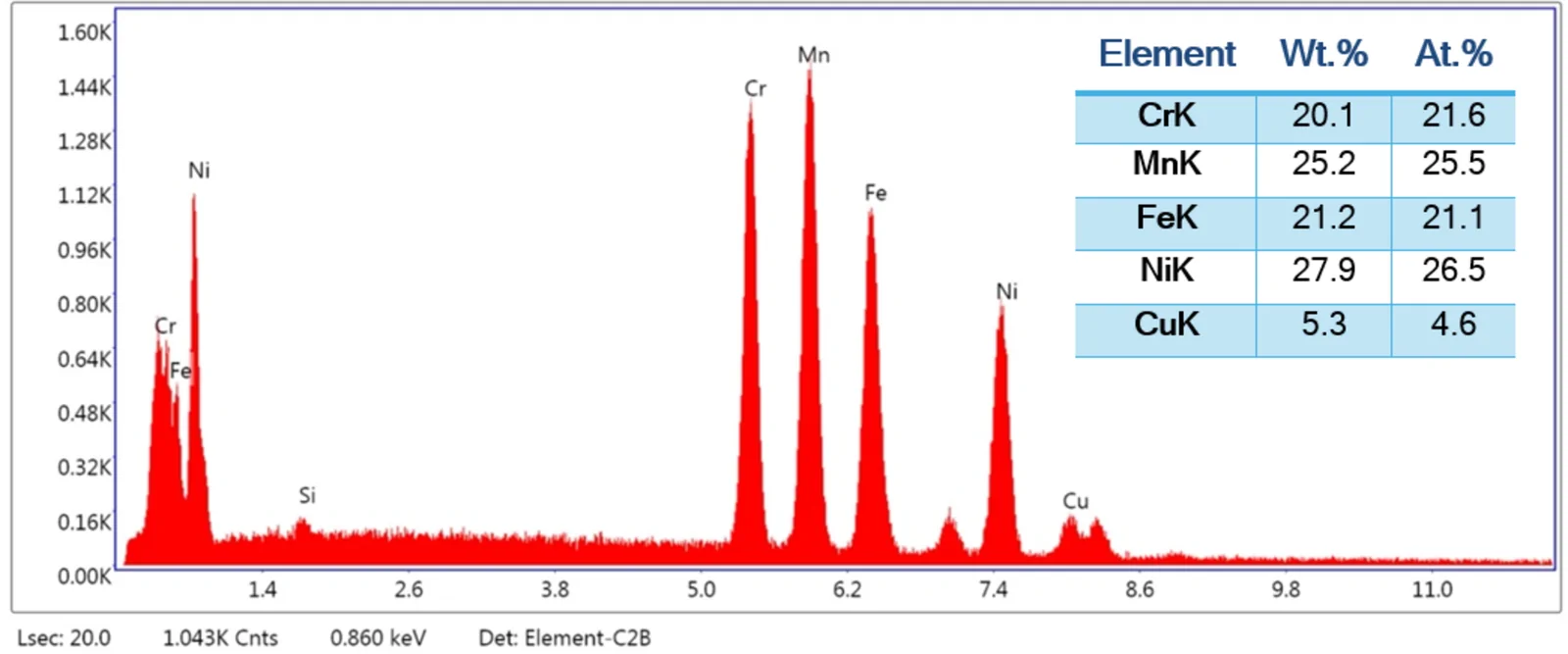
Representative EDS spectrum acquired from the surface of the Cu–Cr–Fe–Mn–Ni HEA coating (Sample 1).

The elemental composition of the HEA coating samples, analyzed by EDS over selected surface areas, is summarized in Table 2. An example of an EDS spectrum acquired from a representative surface region of Sample 1 is shown in Fig. 3, confirming the presence of all principal alloying elements without detectable contamination.

**Fig. 4:**
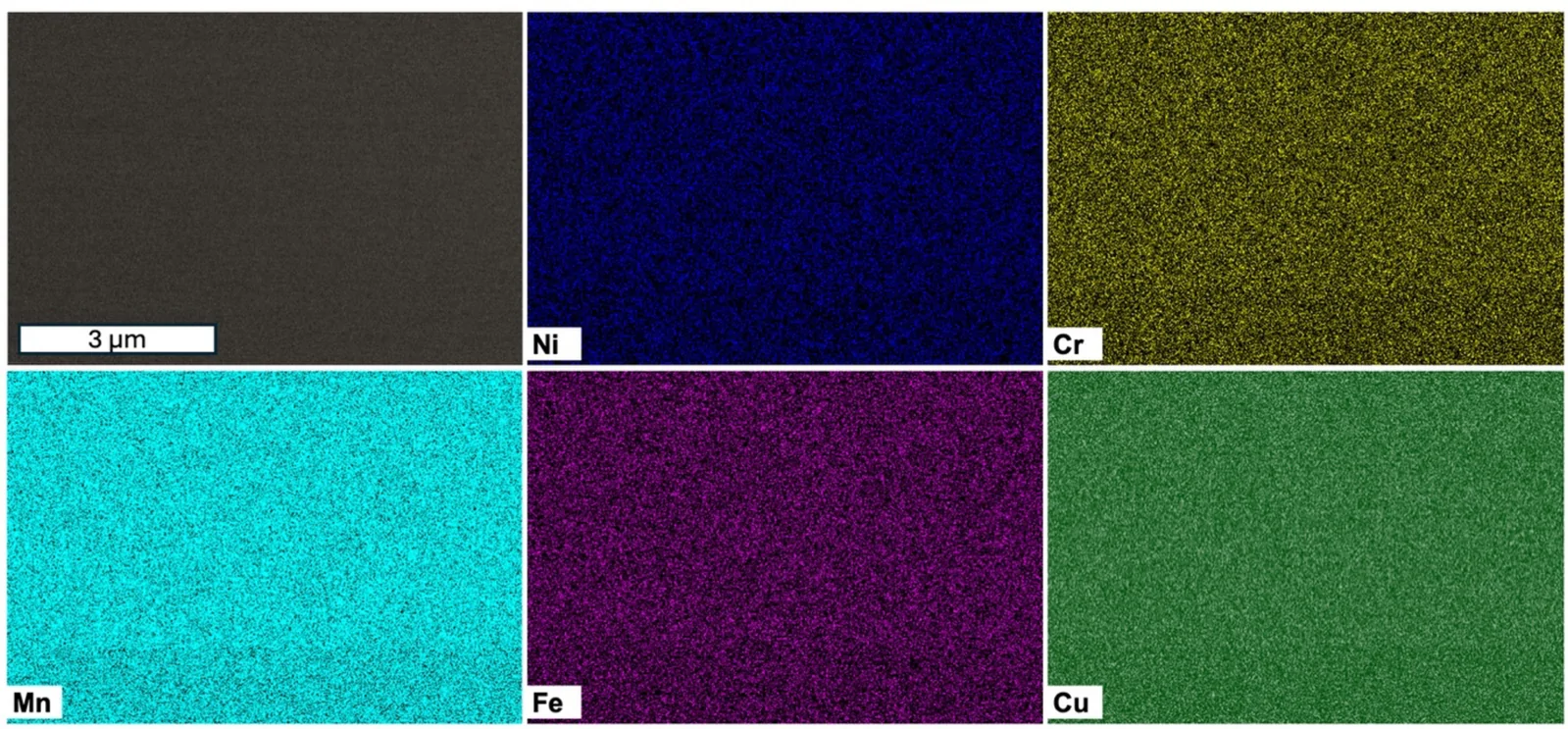
Electron image and corresponding EDS elemental maps of the Cu–Cr–Fe–Mn–Ni HEA coating with 10 at% Cu (Sample 2).

To further assess the spatial distribution of the constituent elements, representative EDS elemental maps of the 10% Cu HEA coating surface are shown in Fig. 4. The elemental maps indicate a relatively uniform distribution of Cu, Cr, Fe, Mn, and Ni within the spatial resolution of the EDS technique and settings, suggesting good compositional homogeneity of the deposited coating.

**Table 2:** Elemental composition of Cu-Cr-Fe-Mn-Ni HEA coatings.

Name	Cu, at%	Cr, at%	Fe, at%	Mn, at%	Ni, at%
Sample 1	4.7	21.7	21.2	25.7	26.7
Sample 2	10.3	23.6	20.6	23.1	22.4

**Fig. 5:**
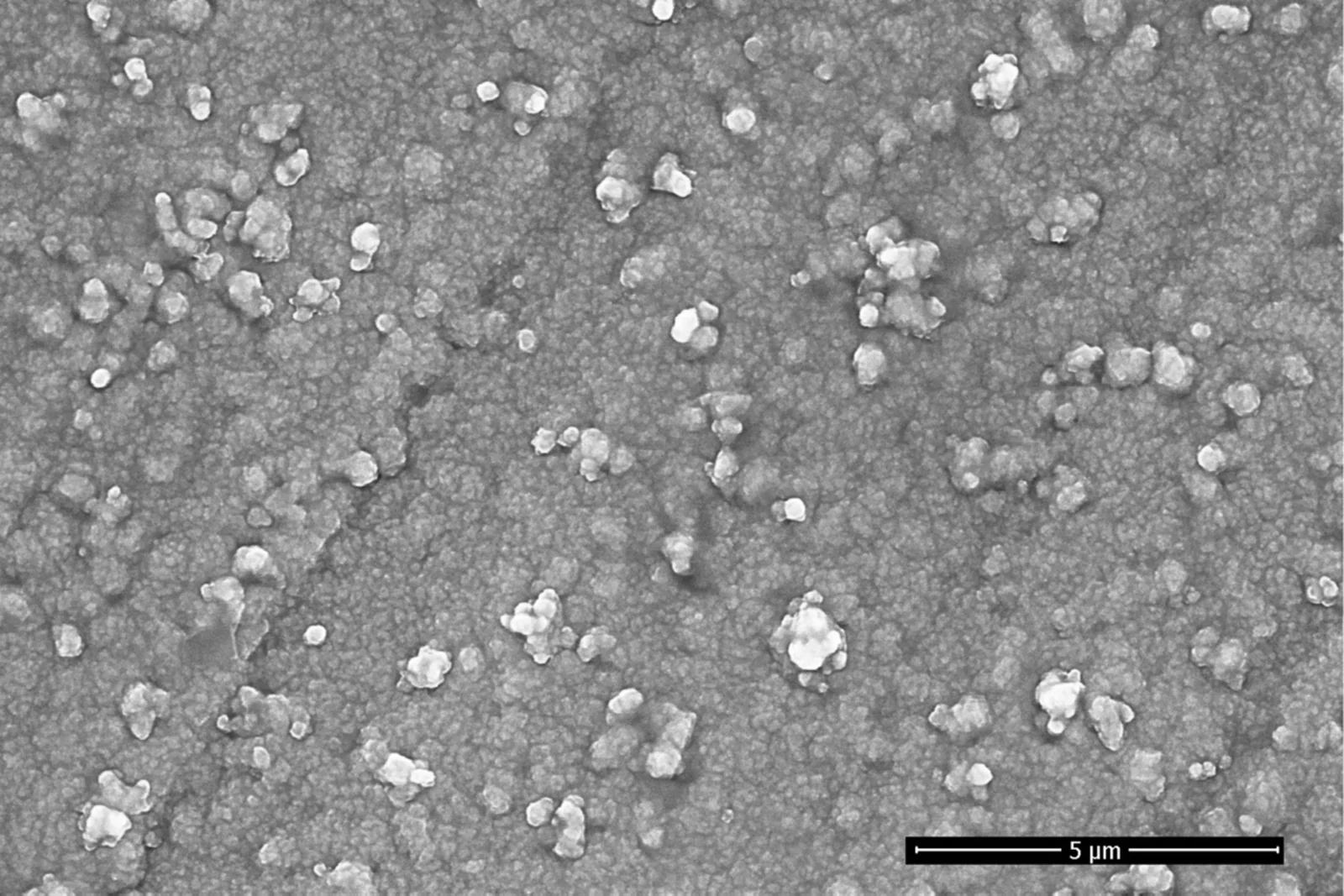
SEM micrograph of the Cu-Cr-Fe-Mn-Ni HEA coating, Sample 2, showing the overview surface morphology. Feature-size analysis is provided in Supplementary Fig. S1.

SEM surface observations of the Cu-Cr-Fe-Mn-Ni HEA coatings reveal a dense and continuous coating layer without visible microcracks or open porosity, as shown in Fig. 5, indicating good coating integrity. SEM images were acquired in secondary electron (SE) mode, and the surface morphology is characterized by a fine, pebbly texture with dispersed bright-contrast features distributed across the surface. The SEM-based image analysis showed a representative apparent surface feature size of ~ 0.12 μm, with most equivalent circular diameters concentrated between 0.07 and 0.17 μm. This supports the formation of a fine nanocrystalline/ultrafine surface morphology, consistent with the dense coating structure observed in the SEM micrographs. The representative segmentation overlay and corresponding size distribution are provided in Supplementary Fig. S1.

The use of an elevated substrate temperature of 400 °C during deposition provided sufficient atomic mobility to promote the formation of a crystalline coating with a refined structural scale, while limiting excessive coarsening during growth. The fine structural scale observed in the coating morphology is generally considered beneficial for mechanical performance. In particular, a nanostructured morphology is expected to contribute to increased hardness and strength through Hall–Petch-type strengthening mechanisms^29,30^, as reported in previous studies^31,32^.

Distinct contrast variations are observed within the SEM micrograph. While such contrast can arise from compositional differences between elements with different atomic numbers, the bright features observed here may also be attributed to surface topography. Elevated surface features, such as partially melted particles or resolidified droplets formed during the deposition process, can appear brighter in secondary electron imaging due to enhanced secondary electron emission. This interpretation is consistent with the rapid solidification conditions associated with electron-beam deposition.

Regardless of the exact nature of the bright features, the resulting coating structure is dense and highly refined. This microstructure, characterized by fine surface features and potential nanoscale phase dispersion, is expected to enhance the coating’s mechanical performance, including hardness and wear resistance, as well as its corrosion resistance, through solid-solution strengthening, grain-refinement effects, and possible precipitation- or phase-related strengthening mechanisms.

**Fig. 6:**
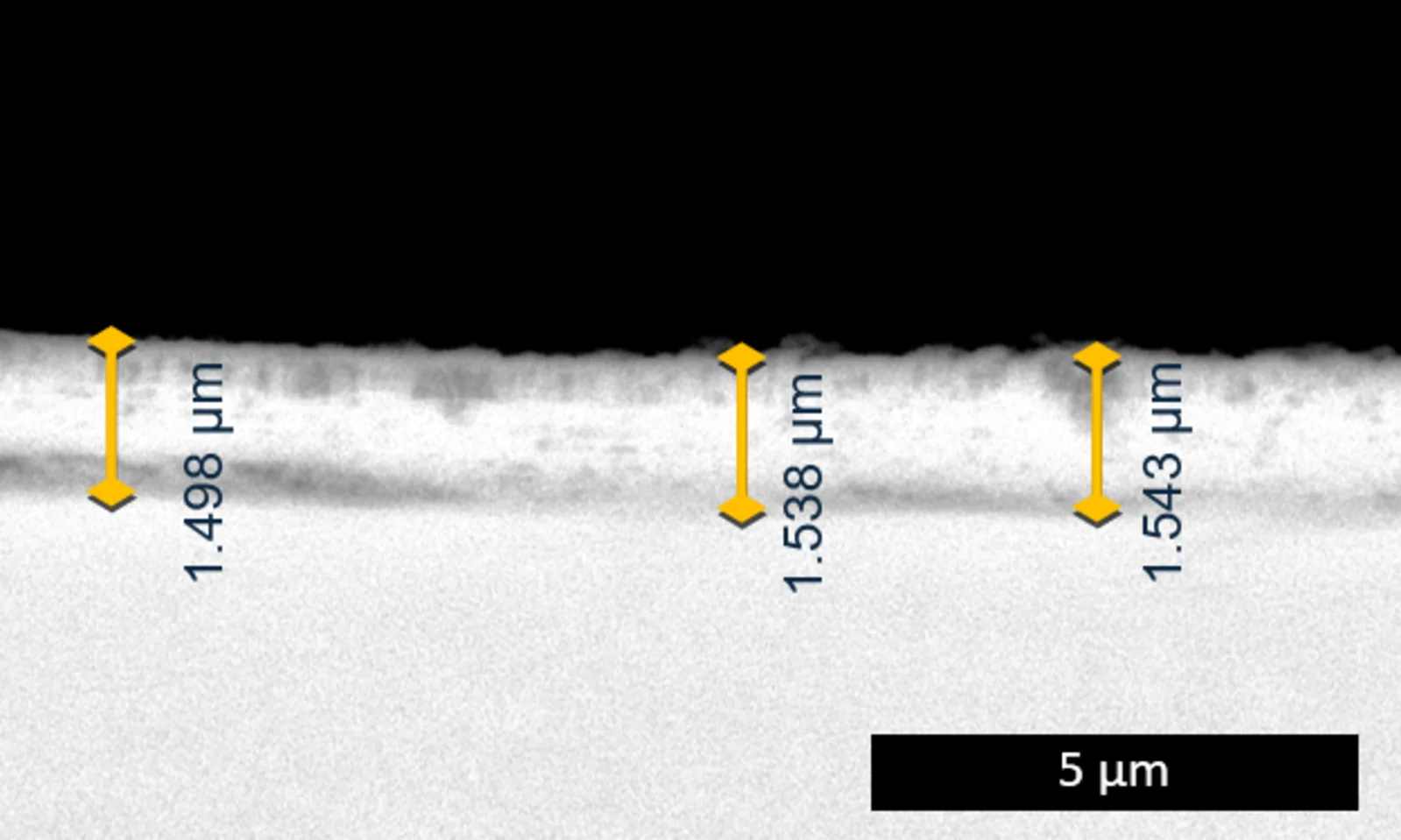
Cross-sectional SEM image of the Cu–Cr–Fe–Mn–Ni HEA coating, Sample 2, showing a continuous coating layer with an average thickness of approximately 1.5 μm.

A representative cross-sectional SEM image of the coating is shown in Fig. 6, confirming the formation of a continuous and uniform coating layer with an average thickness of approximately 1.5 μm. While fine interfacial details cannot be resolved at the available SEM resolution, the image demonstrates good coating continuity across the substrate for both samples. This thickness is commonly reported for HEA coatings deposited by physical vapor deposition techniques^33^ and is sufficient for effective surface protection, while also being suitable for nanoindentation testing by minimizing substrate influence.

### Structural analysis of coatings

**Fig. 7:**
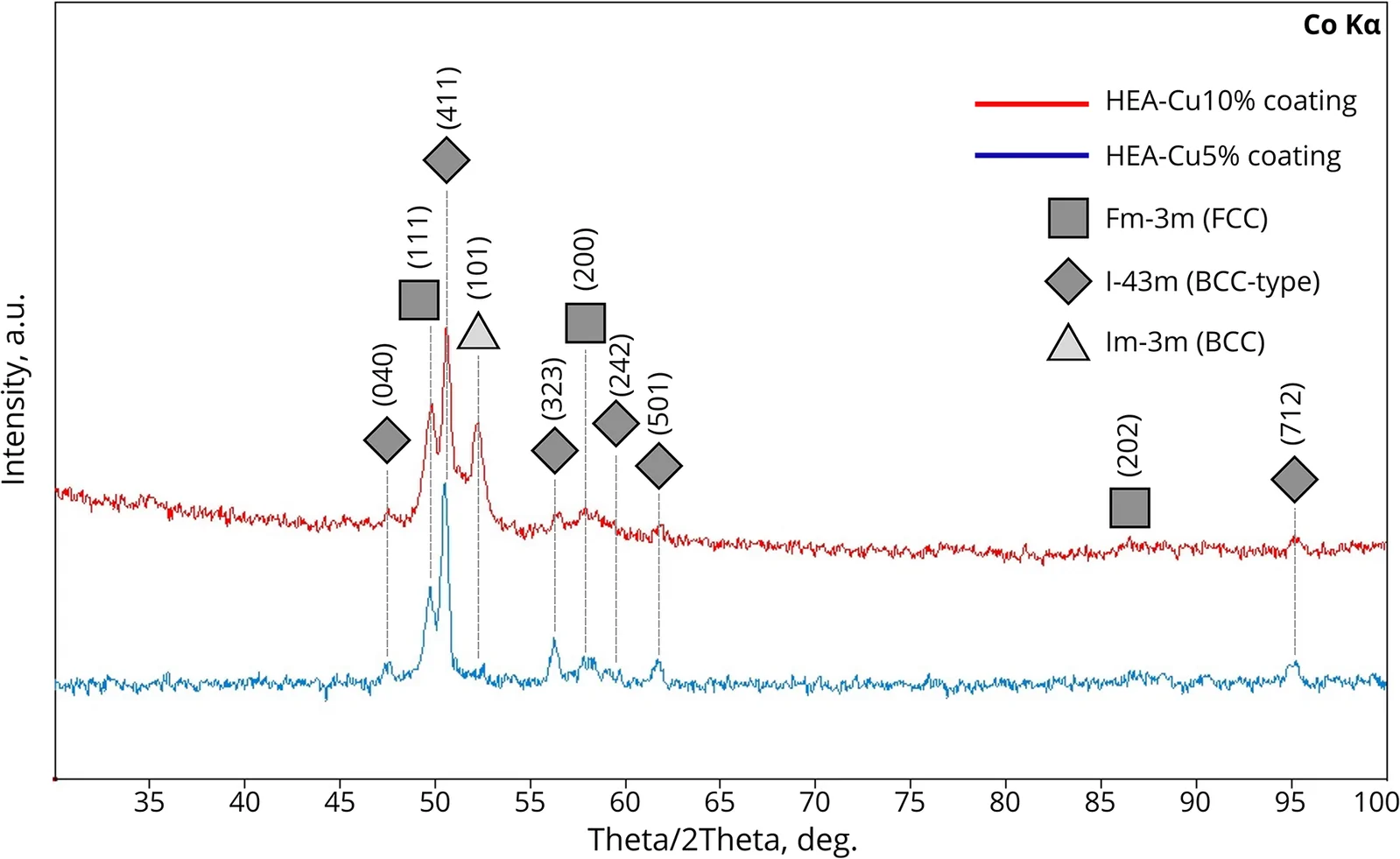
XRD patterns of Cu–Cr–Fe–Mn–Ni HEA coatings with 5 and 10 at% Cu measured using Co Kα radiation, showing FCC- and BCC-type reflections.

XRD analysis of the Cu_x_-Cr-Fe-Mn-Ni HEA coatings revealed the presence of multiple crystalline phases (Fig. 7). The diffraction pattern indicates a dominant cubic structure indexed to the *I-43 m* space group, characterized by the highest peak intensities among the detected reflections. This phase corresponds to an α-Mn–type complex cubic structure, which has been reported in Cr- and Mn-rich HEAs and is sometimes described as χ-like due to its similar diffraction features^34^. This suggests that the coating predominantly crystallized into a non-centrosymmetric cubic lattice derived from a body-centered structure. In contrast to the bulk alloys, where the FCC phase is dominant, the coating exhibits a more complex multiphase constitution, indicating that the non-equilibrium conditions associated with electron-beam physical vapor deposition favor stabilization of metastable or compositionally partitioned phases. In addition, peaks corresponding to the *Fm-3 m* phase were observed, confirming the coexistence of a face-centered cubic (FCC) solid-solution, while weaker reflections indexed to the *Im-3 m* space group indicate the formation of a secondary body-centered cubic (BCC) phase. The coexistence of these three structures, *I-43 m*, *Fm-3 m*, and *Im-3 m*, reflects the complex atomic interactions and elemental partitioning inherent to multicomponent HEA systems, where differences in atomic size and mixing enthalpy promote multiple lattice stabilizations during solidification. The use of electron-beam deposition with a substrate temperature of 400 °C likely enhanced atomic mobility during film growth, promoting stabilization of the complex α-Mn–type phase alongside FCC and BCC solid solutions. No diffraction peaks corresponding to σ- or B2-type intermetallics were detected^35^, indicating that the alloy consists mainly of solid-solution phases. The absence of additional ordered intermetallic phases suggests that phase formation in the coatings is governed primarily by solid-solution stabilization and kinetic effects rather than equilibrium intermetallic precipitation.

### Mechanical properties of HEA coatings

**Fig. 8:**
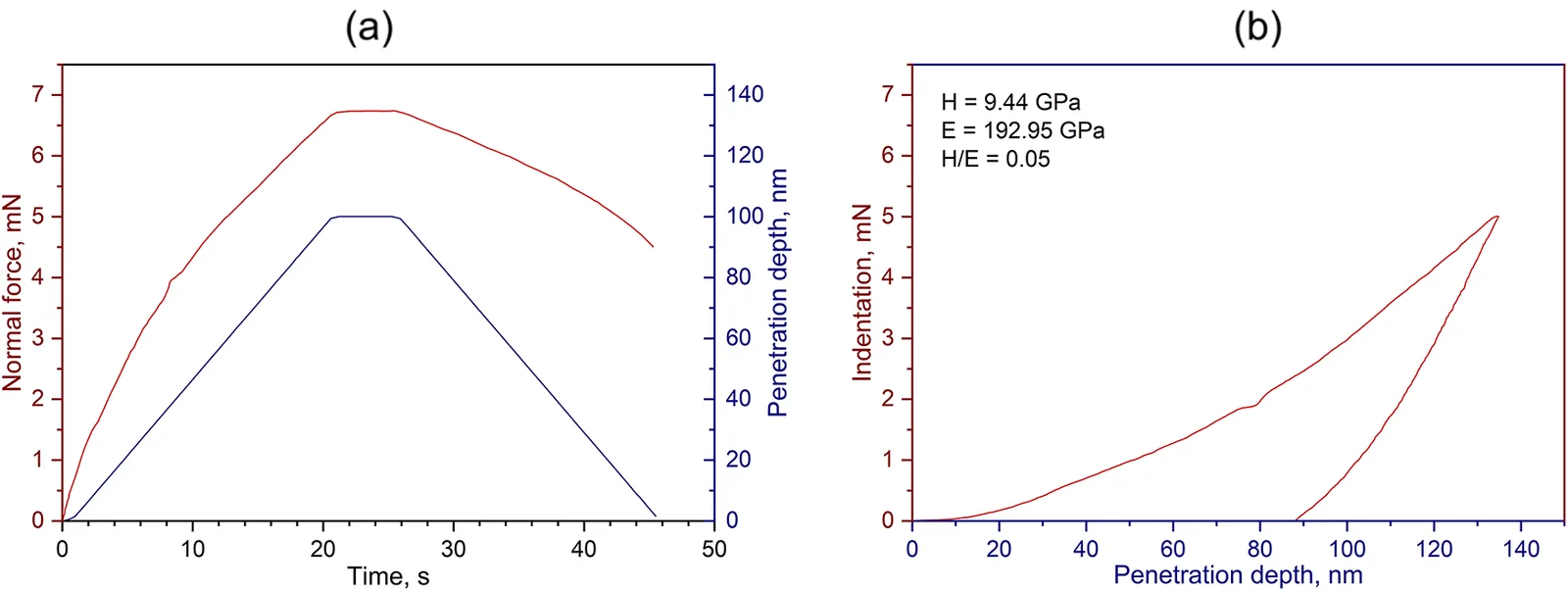
Nanoindentation analysis for Cu–Cr–Fe–Mn–Ni high-entropy alloy coating with 10 at% Cu (Sample 2): (**a**) Force and Penetration Depth vs. Time; (**b**) Load-Displacement Curve.

The nanoindentation results obtained for the investigated HEA coatings showed comparable mechanical response, with no pronounced differences observed within the experimental scatter. The average hardness and elastic modulus were determined as H = 9.44 GPa and E = 192.95 GPa, respectively. Representative nanoindentation data for the Cu10 coating (Sample 2) are shown in Fig. 8; similar load–displacement behavior was observed for the Cu5 coating.

The calculated H/E ratio of approximately 0.05 is considered relatively high for bulk metallic alloys, including HEAs. This parameter is commonly used as an indicator of a material’s ability to elastically accommodate applied stress prior to the onset of plastic deformation and has been widely correlated with resistance to wear and contact damage in metallic materials and coatings^32,36,37^. Accordingly, a higher H/E ratio suggests improved tolerance to elastic strain and enhanced tribological performance, particularly under abrasive or sharp-contact loading conditions. These characteristics indicate that the investigated HEA coatings possess promising potential for high-performance protective applications, including improved resistance to mechanical damage and enhanced durability under long-term service conditions.

### Corrosion resistance

**Fig. 9:**
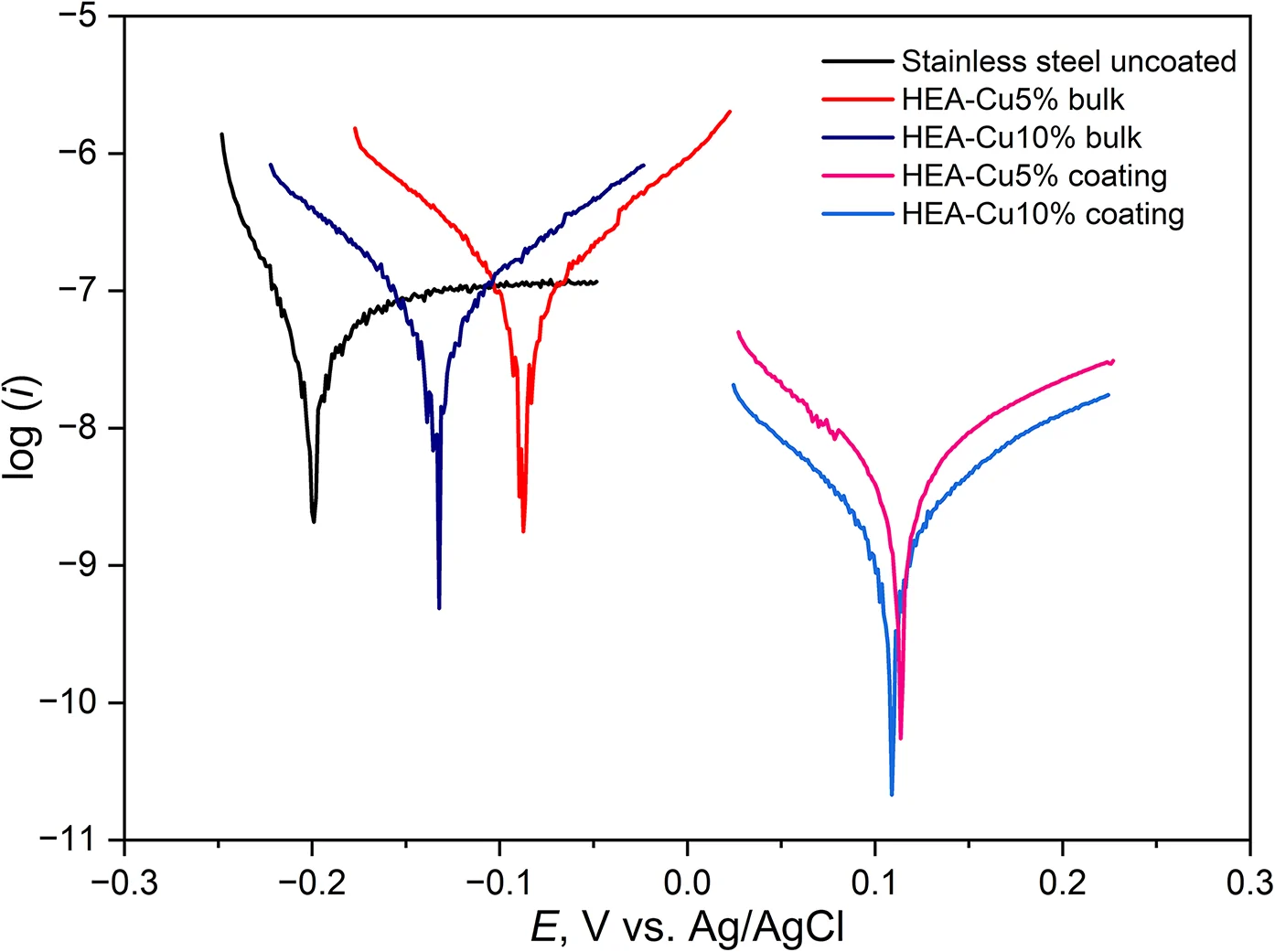
Potentiodynamic polarization curves of uncoated 304 L stainless steel, bulk Cu-Cr-Fe-Mn-Ni HEAs, and Cu-Cr-Fe-Mn-Ni HEA coatings with 5 and 10 at% Cu measured in 35 g/L NaCl at room temperature.

The corrosion behavior of CuCrFeMnNi HEA, both as bulk material and as coatings on 304 L stainless steel, was assessed in comparison to uncoated SS. The potentiodynamic polarization curves are presented in Fig. 9, and the extracted electrochemical parameters are summarized in Table 3.

**Table 3:** Corrosion parameters of Cu_x_-Cr-Fe-Mn-Ni HEA in bulk form and as coatings on 304 L stainless steel, compared to uncoated SS.

Material	E_corr_, mV	j_corr_, nA/cm²	*R*_*p*_, MΩ	i_corr_, nA	Corrosion rate, µm/year
HEA-Cu5 coating	113.5 ± 14.9	7.1 ± 0.4	3.69 ± 0.22	7.1 ± 0.4	0.081 ± 0.005
HEA-Cu10 coating	107.5 ± 10.4	3.4 ± 0.3	7.76 ± 0.71	3.4 ± 0.3	0.039 ± 0.003
HEA-Cu5 bulk	-87.7 ± 7.9	164.2 ± 16.9	0.16 ± 0.02	164.2 ± 16.9	1.91 ± 0.20
HEA-Cu10 bulk	-132.8 ± 23.4	111.4 ± 15.2	0.23 ± 0.03	111.4 ± 15.2	1.29 ± 0.18
SS uncoated	-199.4 ± 22.8	102.1 ± 8.1	0.26 ± 0.02	102.1 ± 8.1	1.19 ± 0.09

The HEA coatings showed markedly improved corrosion resistance compared with both bulk HEAs and uncoated SS, as evidenced by their lower corrosion current densities, higher polarization resistance, and lower corrosion rates. The HEA-Cu5 coating exhibited a corrosion rate of 0.08 μm/year, while the HEA-Cu10 coating showed an even lower rate of 0.04 μm/year. This trend is also reflected in the lower *j*_*corr*_ values of 7.1 and 3.4 nA/cm^2^, respectively, and in the higher *R*_*p*_ values of 3.7 and 7.8 MΩ.

At the same time, bulk HEA samples showed higher corrosion rates than HEA coatings, but still good corrosion resistance comparable to uncoated SS under the same testing conditions. The corrosion rates were 1.91 and 1.29 μm/year for 5% and 10% Cu bulk HEAs, compared with 1.19 μm/year for uncoated SS. The bulk HEA samples also exhibited more positive *E*_*corr*_ values than uncoated SS, indicating a more noble electrochemical response. However, *E*_*corr*_ alone should not be considered a direct indicator of corrosion resistance; therefore, the comparison should be based primarily on *j*_*corr*_, *R*_*p*_, and corrosion rate.

The superior performance of the coatings compared to bulk alloys can be attributed to their dense and refined microstructure, reduced defect density, and the formation of more homogeneous and stable surface oxide layers during deposition. These features enhance polarization resistance and suppress corrosion current density. Among the coated samples, the HEA-Cu10 coating showed the best overall corrosion performance, with the lowest *j*_*corr*_ and corrosion rate and the highest *R*_*p*_.

These findings suggest that HEAs can achieve corrosion performance comparable to or superior to that of SS, while also offering the possibility of functionalization with additional properties, such as antimicrobial activity, representing a promising route toward multifunctional protective materials for various applications.

### Antimicrobial performance

**Fig. 10:**
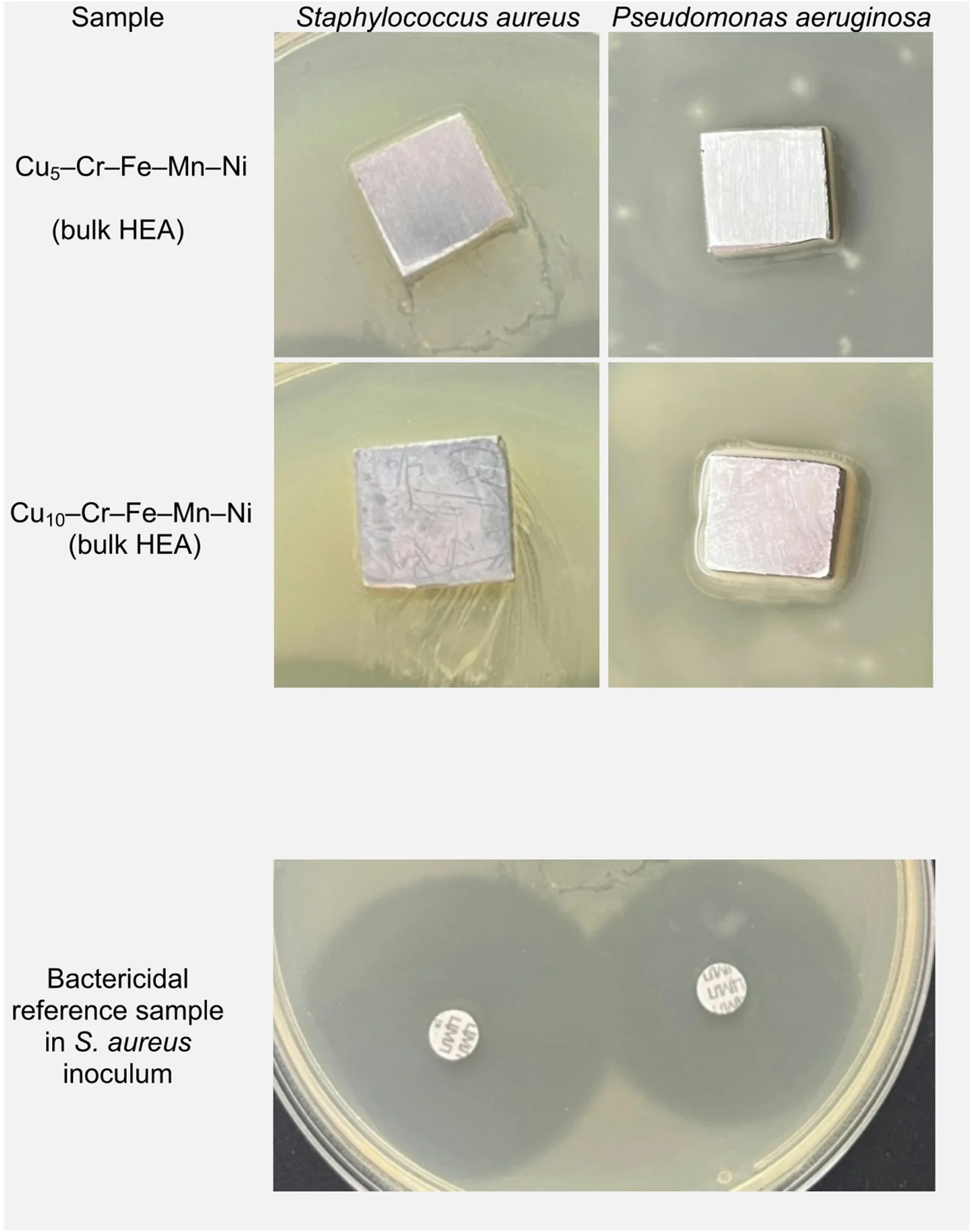
Agar diffusion test showing bacterial growth around Cu_x_–Cr–Fe–Mn–Ni HEA samples and a bactericidal reference; no inhibition halos are observed for the HEAs, while the reference shows clear antibacterial activity.

The antimicrobial performance of Cu_x_–Cr–Fe–Mn–Ni HEA bulk samples was evaluated against *S. aureus* and *P. aeruginosa*. The presence or absence of bacterial growth inhibition around the samples was examined after incubation, and representative photographs of bacterial growth are presented in Fig. 10.

It was found that the HEA materials did not exhibit significant antibacterial activity against the Gram-positive *S. aureus* or the Gram-negative P. *aeruginosa* under the present experimental conditions. No distinct inhibition halos were observed around either HEA sample for both *S. aureus* and *P. aeruginosa*, in contrast to the bactericidal reference, which exhibited a clear zone of growth inhibition. This indicates the absence of detectable diffusion-mediated antibacterial activity in the agar diffusion test. It should be noted that this qualitative method is primarily sensitive to antimicrobial effects associated with the release and diffusion of active species, such as metal ions, into the surrounding medium. Consequently, potential surface-contact-dependent antibacterial mechanisms, which have been reported for several metallic and HEA systems, are not fully captured by this test.

In the next stage, an additional method, the time-kill assay, was employed to assess the antimicrobial effect as a function of the exposure time between the material and the target microorganisms. The time-kill test ensures uniform contact between the samples and bacterial cells, allowing for a more accurate evaluation of bacterial viability reduction. Unlike the agar diffusion method, this approach generally reveals a higher number of positive cases of antimicrobial activity (75 cases compared to 24 for the agar-based test).

**Fig. 11:**
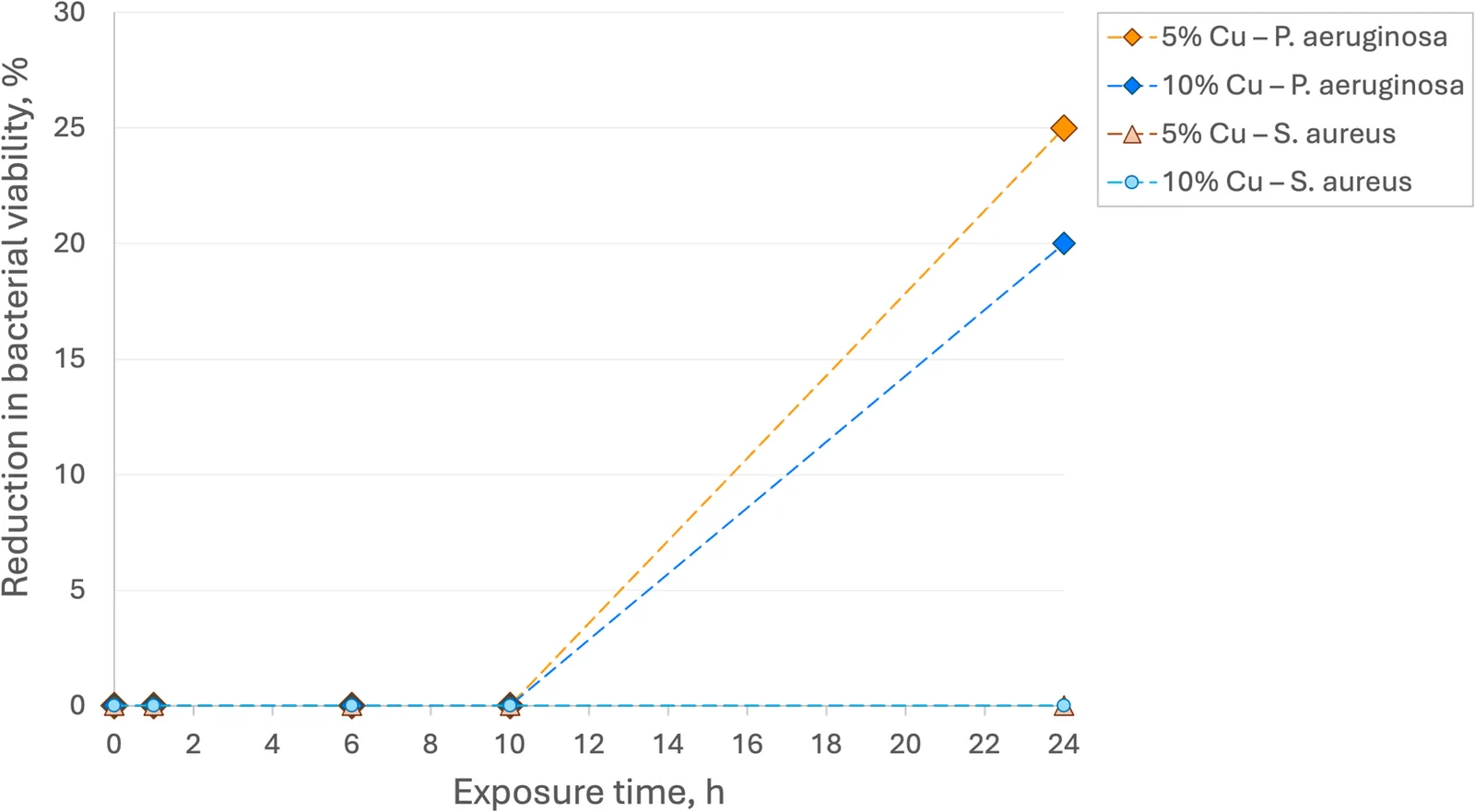
Antimicrobial effect of Cu_x_-Cr-Fe-Mn-Ni HEAs on *S. aureus* and *P. aeruginosa* over 1–24 h incubation.

The bactericidal activity of the samples determined by this method is summarized in Fig. 11. According to the results, the materials exhibited moderate antibacterial activity only against *P. aeruginosa*. While no significant decrease in the number of *P. aeruginosa* cells was observed during the first 10 h of incubation, a noticeable reduction in viable bacterial counts occurred after prolonged co-culturing with the HEA samples. After 24 h, bacterial viability decreased by approximately 20–25%.

As noted previously, no antimicrobial effect was detected against the Gram-positive *S. aureus*, whereas a measurable long-term inhibitory effect was observed against the Gram-negative *P. aeruginosa*. This difference can be rationalized based on well-established structural distinctions between Gram-positive and Gram-negative bacterial cell envelopes. Gram-positive bacteria such as *S. aureus* possess a thick, highly cross-linked peptidoglycan layer, which can limit the penetration and interaction of metallic ions or reactive species with the cytoplasmic membrane. In contrast, Gram-negative bacteria such as *P. aeruginosa* have a thinner peptidoglycan layer and an additional outer membrane containing lipopolysaccharides, which may facilitate different interactions with metal ions and metallic surfaces. Such differences have been widely reported to influence bacterial susceptibility to metallic and Cu-containing antimicrobial materials^8,38–40^.

The agar diffusion and time–kill assays were performed on bulk HEA alloys, whereas the long-term microbiologically influenced corrosion experiments described below were conducted on HEA coatings deposited on 304 L stainless steel substrates. Owing to differences in phase constitution and microstructure between the bulk alloys and the coatings, variations in antimicrobial and corrosion-related behavior may be expected.

**Fig. 12:**
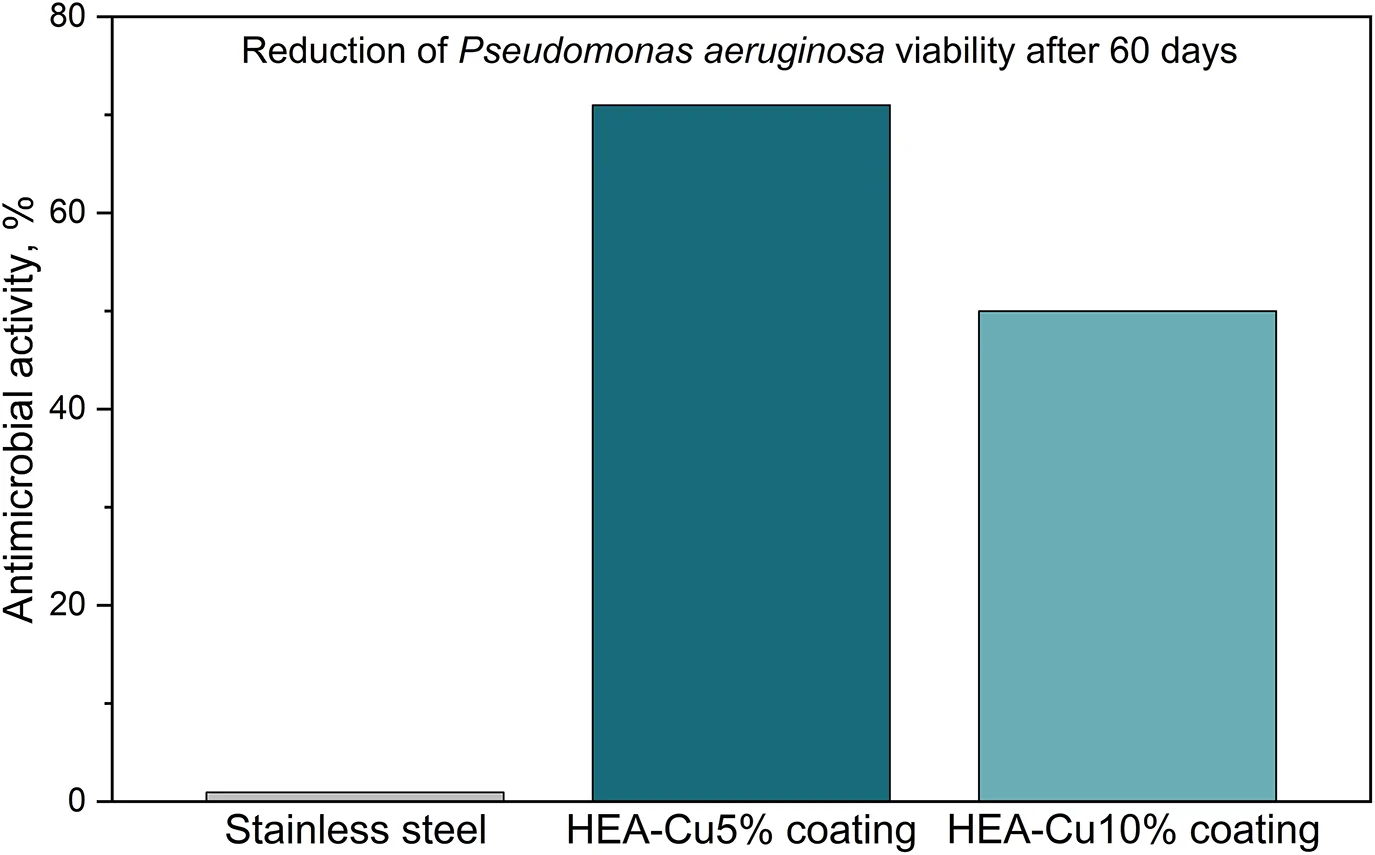
Antimicrobial activity of Cu-Cr-Fe-Mn-Ni HEA coatings against *Pseudomonas aeruginosa*, expressed as bacterial viability reduction after 60 days of exposure in 3.5% NaCl solution.

The antimicrobial and corrosion-related behavior of Cu-Cr-Fe-Mn-Ni coatings on stainless steel was evaluated after 60 days of exposure in 3.5% NaCl saline solution, both under abiotic and biotic conditions with a *P. aeruginosa* consortium. Table 4 summarizes bacterial growth, biofilm formation, viable cell counts, pH changes, and coupon surface observations. Figure 12 demonstrates calculated antimicrobial rate, or reduction of bacterial growth, for studied surfaces after 60 days of exposure.

**Table 4:**
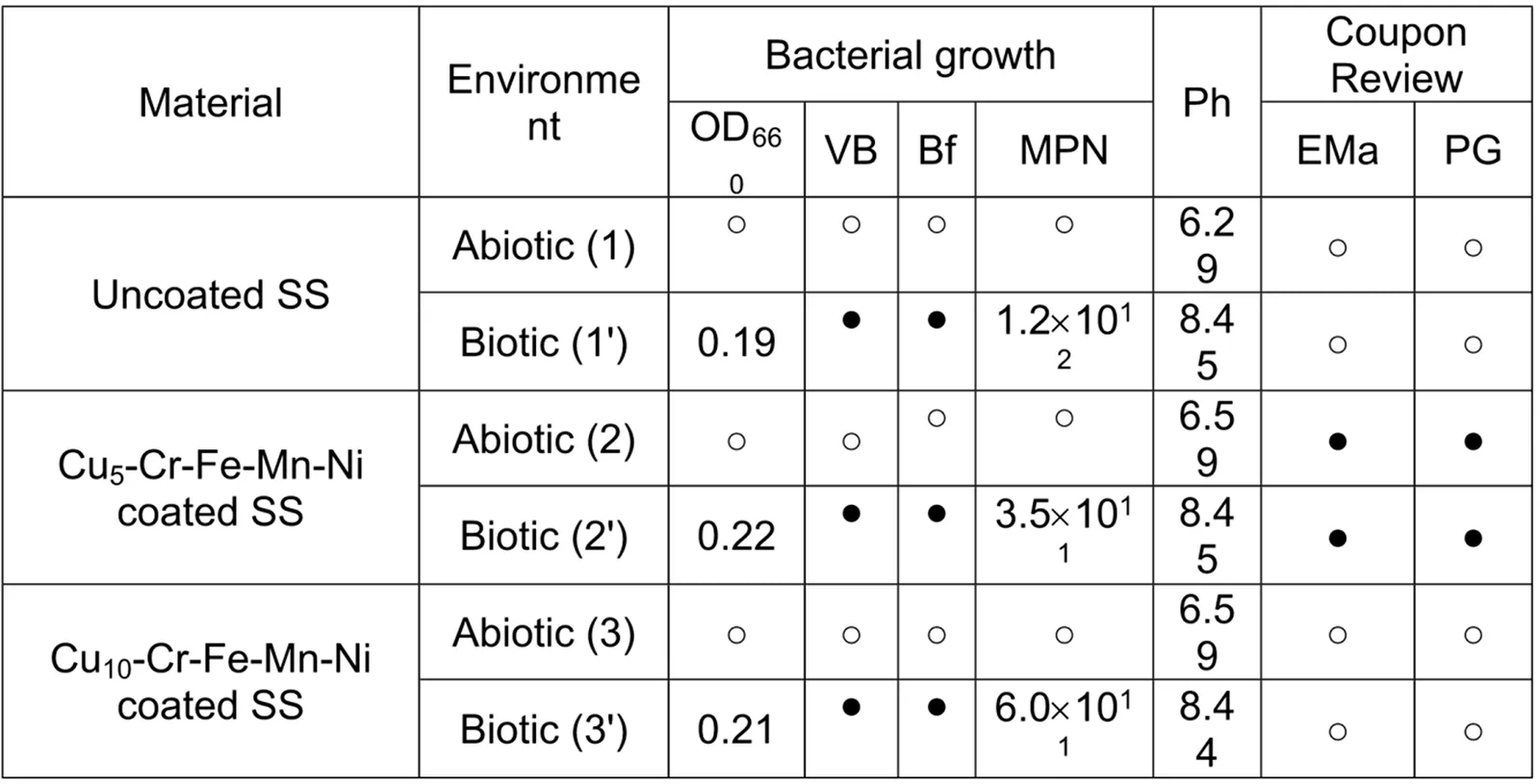
Antimicrobial and corrosion-related effects of Cu-Cr-Fe-Mn-Ni HEA coatings on 304 L stainless steel (SS) after 60 days of exposure to *Pseudomonas aeruginosa*.

OD₆₆₀ – optical density at 660 nm (planktonic bacterial growth), MPN – most probable number of viable bacteria (cells/mL), pH – final pH of the solution, VB – bacterial viability, Bf – biofilm formation, EMa – macroscopic surface changes, PG – physical degradation or delamination of the coating (● = observed, ○ = not observed).

Bacterial growth in the saline solutions was initially estimated by measuring the optical density at 660 nm (OD_660_), which reflects culture turbidity and provides a rapid indication of bacterial cell density. Higher OD_660_ values indicate greater bacterial presence, while lower values correspond to fewer suspended cells. For example, the uncoated 304 L biotic sample exhibited a OD_660_ of 0.19, consistent with dense bacterial growth, whereas the Cu-Cr-Fe-Mn-Ni-coated samples had slightly higher OD_660_ values (0.21–0.22), indicating the presence of bacteria in solution. Viable cell counts measured by MPN confirmed these observations, showing 1.2 × 10^12^ cells/mL for uncoated 304 L and 3.5 × 10^11^ cells/mL for the Cu 5% coating, and 6.0 × 10^11^ cells/mL for the Cu 10% coating. Using these values, the antimicrobial rate of the coatings can be estimated, with Cu 5% achieving ~ 71% reduction compared to uncoated steel, and Cu 10% ~50% reduction.

The pH of the saline solution increased from the initial 5.45 to 8.44–8.45 in biotic samples, reflecting metabolic activity of the *P. aeruginosa* consortium. Abiotic control samples remained near-neutral (6.29–6.59), confirming that the pH rise was biologically induced.

Macroscopic and microscopic inspection of the coupons revealed differences in corrosion resistance. The uncoated stainless steel showed no weight loss or surface damage, indicating excellent corrosion resistance under both abiotic and biotic conditions. The Cu 5% Cu-Cr-Fe-Mn-Ni coating displayed slight delamination and surface irregularities, which may indicate both reduced overall corrosion resistance and lower coating adhesion. In contrast, the Cu 10% coating remained intact with no weight loss or delamination, suggesting better corrosion resistance, likely due to a more stable passive film. Interestingly, this improved passive layer may also reduce copper ion release, explaining the lower antimicrobial effect of Cu 10% compared to Cu 5%.

Overall, the results indicate that CuCrFeMnNi coatings on 304 L stainless steel provide moderate antimicrobial activity against *P. aeruginosa*, with effectiveness influenced by Cu content. Corrosion resistance improves with higher Cu content, but at the cost of reduced ion-mediated antimicrobial action. These observations highlight the trade-off between antimicrobial efficacy and corrosion protection, with the Cu 5% coating showing the highest antimicrobial rate alongside minor surface delamination, and Cu 10% coating demonstrating superior corrosion resistance but a lower antimicrobial effect.

### XPS analysis and surface chemistry of Cu-Cr-Fe-Mn-Ni HEA coatings

**Fig. 13:**
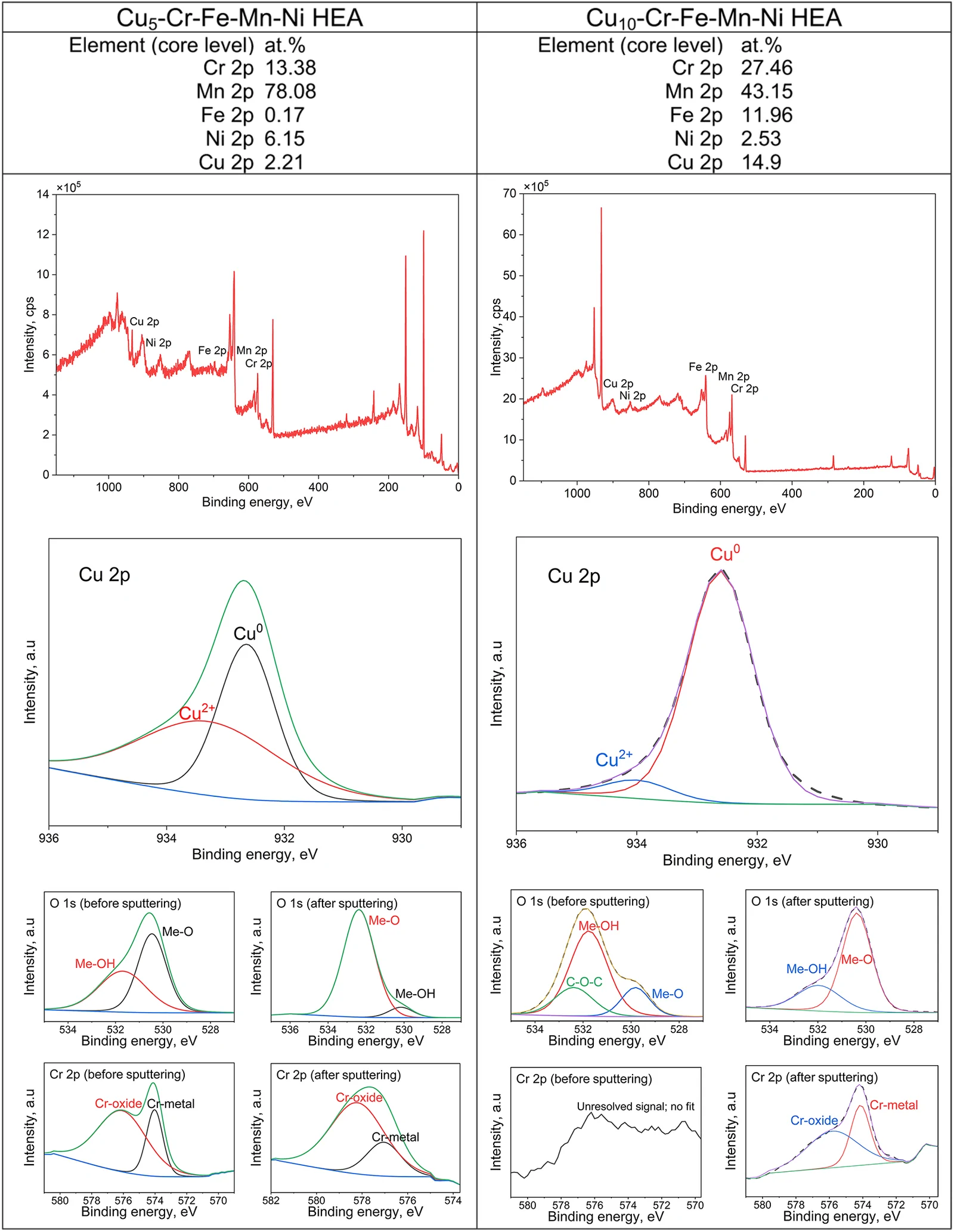
XPS analysis of Cu_x_-Cr-Fe-Mn-Ni HEA coatings for Sample 1 (5 at% Cu) and Sample 2 (10 at% Cu), showing surface composition, survey spectra, O 1s and Cr 2p spectra before and after sputtering, and post-sputtering Cu 2p spectra. The data highlight surface enrichment, oxidation states, and the persistence of the native oxide layer after sputtering.

To better understand the surface composition and oxidation states of the Cu-Cr-Fe-Mn-Ni HEA coatings, X-ray photoelectron spectroscopy (XPS) was performed on samples with 5 at% (Sample 1) and 10 at% Cu (Sample 2). The analysis provides insights into the distribution of alloying elements at the surface, the nature of the native oxide layer, and the effects of sputtering, which are critical for interpreting both corrosion resistance and antimicrobial performance. XPS characterization of the Cu-Cr-Fe-Mn-Ni HEA coatings revealed pronounced surface compositional changes depending on Cu content. XPS survey and high-resolution spectra of both samples are presented in Fig. 13.

#### Sample Cu_5_-Cr-Fe-Mn-Ni HEA (5 at% Cu)

The surface composition of the Cu5 HEA coating shows a strong manganese enrichment (78 at% vs. ~ 25 at% nominal) and severe depletion of iron (0.17 at% vs. ~ 25 at% nominal), while chromium, nickel, and copper remain near nominal values. The O 1s spectrum reveals two main components: a dominant peak at ~ 530.2 eV assigned to metal oxides (likely MnO_x_, Cr_2_O_3_, NiO) and a shoulder at ~ 531.8 eV corresponding to hydroxyl species or adsorbed water, indicating a thick, hydrated native oxide layer^41–43^.

After sputtering, the overall O 1s signal intensity decreases substantially, revealing a thinner, predominantly metallic oxide layer (~ 530.1 eV), with minor residual OH groups (~ 531.7 eV). The Mn 2p_3/2_ spectrum shows Mn^3+^ (~ 641.8 eV) as the dominant species, with minor Mn^4+^ (~ 643.0 eV), suggesting a mixture of Mn_2_O_3_ and MnO_2_ or Mn_3_O_4_. Chromium remains mostly as Cr_2_O_3_, confirming a stable, passivating oxide layer^44^. Therefore, sputtering exposes some metallic Cr and Mn beneath the surface, but the oxide persists, consistent with the HEA’s passivation behavior.

#### Sample Cu_10_–Cr–Fe–Mn–Ni HEA (10 at% Cu)

Increasing Cu content to 10 at% alters the surface composition: Mn enrichment decreases to 43 at%, Fe partially recovers to 12 at%, Ni is slightly depleted, and Cu becomes significantly enriched (14.9 at% vs. nominal 10%), indicating strong surface segregation. Given the similar bulk Fe content in both alloys, this significant Fe recovery indicates a substantially thinner Mn-rich oxide overlayer on Cu10. The added copper thus suppresses the growth of this thick scale, enabling photoelectron detection from the underlying metal. The addition of 10 at% Cu fundamentally alters the surface architecture: it suppresses the formation of a thick Mn-rich oxide overlayer, leading to a thinner mixed oxide scale and significant surface enrichment of Cu. Concurrently, the dominant manganese oxidation state shifts from Mn^3+^ in the Cu5-HEA to Mn^4+^ in the Cu10-HEA. This redox transition, indicative of a strong Cu-Mn surface interaction where copper stabilizes the higher manganese oxidation state, is consistent with mechanisms reported for Cu-modified manganese oxides^45,46^.

The O 1s spectrum shows a mixed metal oxide layer, now including significant contributions from Cu (Cu^2+^/Cu^+^), Mn, Cr, and Fe. In the Cu10 sample, the Cr 2p spectrum before sputtering was unresolved and could not be reliably fitted, most likely because the Cr signal was masked by the Cu/Mn-enriched native oxide layer. Before sputtering, a native oxide layer (> 3 nm) attenuated the metallic coating signals. After sputtering, only a residual mixed oxide/metallic layer remained. The thickness of this residual oxide was estimated to be ~ 0.6 nm, corresponding to the deepest inner part of the native oxide layer directly adjacent to the underlying metallic HEA. At this stage, the Cr 2p signal became clearly distinguishable, allowing separation of Cr-metal and Cr-oxide components. Cr^3+^ dominates the oxide contribution (~ 70–80%), while Mn is mostly present as Mn^4+^ (MnO_2_ or oxidized Mn_3_O_4_), and Cu primarily as Cu^+^ (Cu_2_O). The sharper Cu peak reflects both its higher surface concentration and the cleaner sputtered surface.

#### Correlation with corrosion and antimicrobial behavior

The XPS results provide a direct explanation for the observed performance trade-off between the two Cu_x_-HEA coatings compositions. The superior corrosion resistance of the Cu10 coating, evidenced by its lower corrosion rate and higher polarization resistance (Table 3), is attributed to the formation of a more uniform and stable passive layer composed of a protective mixture of Cr_2_O_3_ and Cu_2_O^44,47^. Conversely, the higher antimicrobial rate (~ 71%) of the Cu5 coating against *P. aeruginosa* (Fig. 11) is linked to its surface chemistry. The Mn-rich, hydrated oxide layer appears less protective, facilitating a higher release rate of biocidal Cu^2+^ ions. The inherent instability of Mn(III) species in the oxide lattice drives this enhanced release. Specifically, Mn^3+^ undergoes aqueous disproportionation (2Mn^3+^ → Mn^4+^ + Mn^2+^). This reaction triggers a structural transformation that expels incorporated Cu ions into the solution^46^. This explains its superior antimicrobial efficacy despite its lower Cu concentration. Thus, XPS confirms that surface composition and oxidation states directly influence both corrosion resistance and antimicrobial efficacy in studied Cu-containing HEAs.

A comparative summary of the multifunctional performance and structure–property relationships discussed above, illustrating the interplay between phase composition, surface chemistry, mechanical properties, corrosion resistance, and antimicrobial activity, is presented in Table 5.

**Table 5:** Comparative summary of the structure-property relationships and multifunctional performance of Cu_x_-Cr-Fe-Mn-Ni HEAs and coatings.

Property	Bulk HEA	E-Beam Coating	Key Insights / Trends
Microstructure & phase composition	Predominantly FCC with minor BCC; dendritic morphology	Nanocrystalline/ultrafine morphology with SEM-resolved grains centered around ~ 0.12 μm; complex phase coexistence (I-43 m, FCC, BCC)	Cu content influences BCC fraction and phase distribution; coatings exhibit dense, defect-free microstructure
Mechanical properties	Hardness: 240–290 HV	Hardness: ~9.4 GPa; elastic modulus: ~193 GPa; H/E = 0.05	Higher Cu → higher hardness; nanocrystalline coating enhances wear resistance and plastic deformation tolerance
Corrosion resistance	Comparable to SS (1.29–1.91 μm/year)	Significantly improved (0.039–0.081 μm/year); high *R*_*p*_ (3.69–7.76 MΩ)	Cu10 coating shows the best corrosion resistance; stable Cr_2_O_3_/Cu_2_O-containing passive layers enhance protection
Antimicrobial activity	Moderate inhibition against *P. aeruginosa*; no effect on *S. aureus*	Cu-dependent: 5 at% Cu → ~71% reduction; 10 at% Cu → ~50% reduction	Trade-off between corrosion protection and antimicrobial activity; surface chemistry controls multifunctionality
Surface chemistry (XPS)	-	Cu5: less protective surface oxide; Cu10: Cu-enriched, more stable Cr_2_O_3_/Cu_2_O-based passive layer	Surface composition and oxide characteristics govern corrosion behavior and antimicrobial response
Applications	Multifunctional protective materials for marine, energy, and industrial environments; balance between corrosion protection and antimicrobial activity can be tuned by Cu content and fabrication/deposition conditions		

## Conclusions

Cu_x_-Cr-Fe-Mn-Ni high-entropy alloys and their E-beam-deposited coatings exhibit a remarkable combination of mechanical strength, corrosion resistance, and tunable antimicrobial activity, highlighting their potential as multifunctional protective materials. Bulk alloys exhibit predominantly FCC structures with minor BCC contributions, dendritic microstructures, and hardness ranging from 240 to 290 HV, while coatings develop dense nanocrystalline/ultrafine structures with apparent surface features centered around ~ 0.12 μm, complex phase coexistence (*I‑43 m*, FCC, BCC), and high hardness (~ 9.4 GPa) with an H/E ratio of 0.05, indicating excellent mechanical properties and potential for improved resistance to wear and contact damage.

Corrosion tests reveal that the HEA coatings provide substantially enhanced corrosion protection compared with both bulk HEAs and uncoated SS. In particular, the HEA-Cu10 coating exhibited the lowest corrosion rate of 0.039 μm/year, together with the lowest corrosion current density and highest polarization resistance. In contrast, the moderate-Cu HEA-Cu5 composition showed a comparatively higher corrosion rate, which may indicate greater surface reactivity and potentially greater availability of Cu-containing species, consistent with the observed approximately 71% reduction in *Pseudomonas aeruginosa* viability.

XPS analysis supports this interpretation by showing that surface composition and oxide characteristics critically govern the trade-off between corrosion protection and antimicrobial efficacy. The formation of a more stable Cr_2_O_3_/Cu_2_O-containing passive layer favors corrosion resistance in the Cu10 coating, whereas the less protective surface oxide in the Cu5 coating may favor the release of Cu-containing species and contribute to enhanced antimicrobial activity.

These findings demonstrate that Cu_x_-Cr-Fe-Mn-Ni HEAs offer tunable multifunctionality, making them highly promising candidates for demanding applications in marine, energy, and industrial environments where both long-term corrosion resistance and antimicrobial performance are required.

## Supplementary Information

Below is the link to the electronic supplementary material.

Supplementary Material 1

## Data Availability

Data will be made available on request.
