# Supplementary material for: Tunable mechanical, corrosion, and antimicrobial performance in multifunctional Cux‑Cr-Fe-Mn-Ni high-entropy alloys and E-beam coatings
Source: Sci Rep. 2026 Jun 14;16:26808. doi: 10.1038/s41598-026-55056-0 (PMC13519015; doi:10.1038/s41598-026-55056-0)
Supplement: Supplementary file 1 [file 41598_2026_55056_MOESM1_ESM.pdf]

Supplementary Information for:

Tunable Mechanical, Corrosion, and Antimicrobial Performance in Multifunctional Cu_x-Cr-Fe-Mn-Ni High-Entropy Alloys and E-Beam Coatings

Bogdan Postolnyi[1,2,3,a)], Dumitru Mitrica[2], Arcadii Sobetkii[2], Aizhan B. Talipova[4], Krzysztof Rokosz[5], Volodymyr Buranych[6], Laurentiu-Florin Mosinoiu[2], Martin Kusý[7], Feodor M. Borodich[8], Vladimir A. Levchenko[9], Laura-Madalina Cursaru[2], Alexander Pogrebnjak[2,3,7,a)]

[1]Institute of Physics for Advanced Materials, Nanotechnology and Photonics, Faculty of Sciences of the University of Porto, 4169-007 Porto, Portugal

[2]National R&D Institute for Non−Ferrous and Rare Metals, 077145 Pantelimon, Romania

[3]Sumy State University, 116 Kharkivska st., 40007 Sumy, Ukraine

[4]Department of Biotechnology, Al-Farabi Kazakh National University, Almaty 050040, Kazakhstan

[5]Division of Surface Electrochemistry & Technology, Faculty of Mechanical Engineering, Koszalin University of Technology, Racławicka 15-17, PL 75-620 Koszalin, Poland

[6]Institute of Electrical Engineering, Slovak Academy of Sciences, Dubravska cesta 9, 84104 Bratislava, Slovakia

[7]Institute of Materials, Faculty of Materials Science and Technology in Trnava, Slovak University of Technology in Bratislava, 917 24, Trnava, Slovakia

[8]College of Aerospace Engineering, Chongqing University, 400030 Chongqing, China

[9]Institute of Advanced Coatings and Materials, Taizhou University, 318000 Taizhou, China

[a)] Authors to whom correspondence should be addressed: b.postolnyi@gmail.com and a.d.pogrebnjak@gmail.com

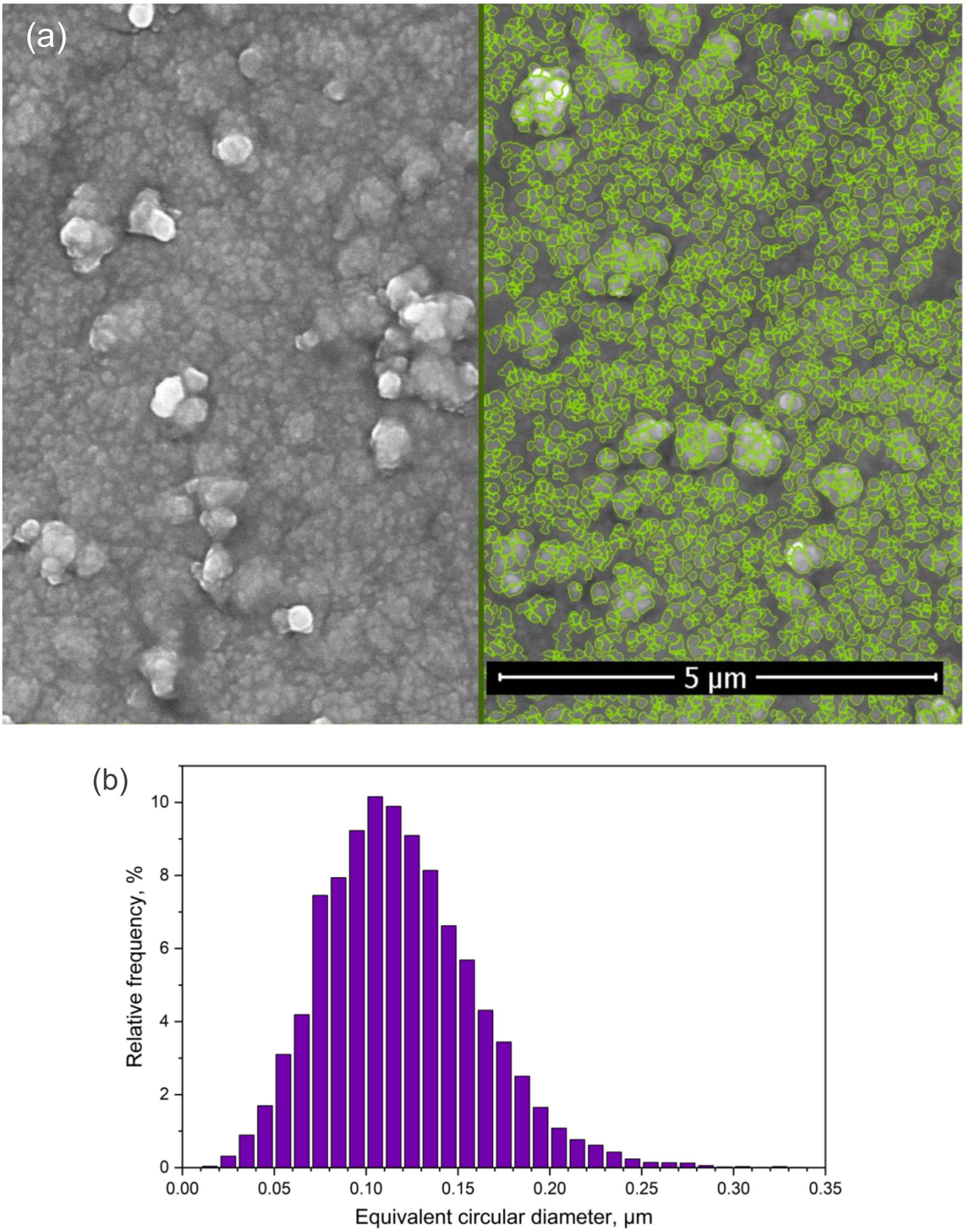

Figure S1. SEM-based apparent grain/feature-size analysis: (a) representative cropped SEM region with overlaid boundaries shown to illustrate the segmentation procedure and (b) equivalent circular diameter distribution calculated from the full analyzed SEM area.

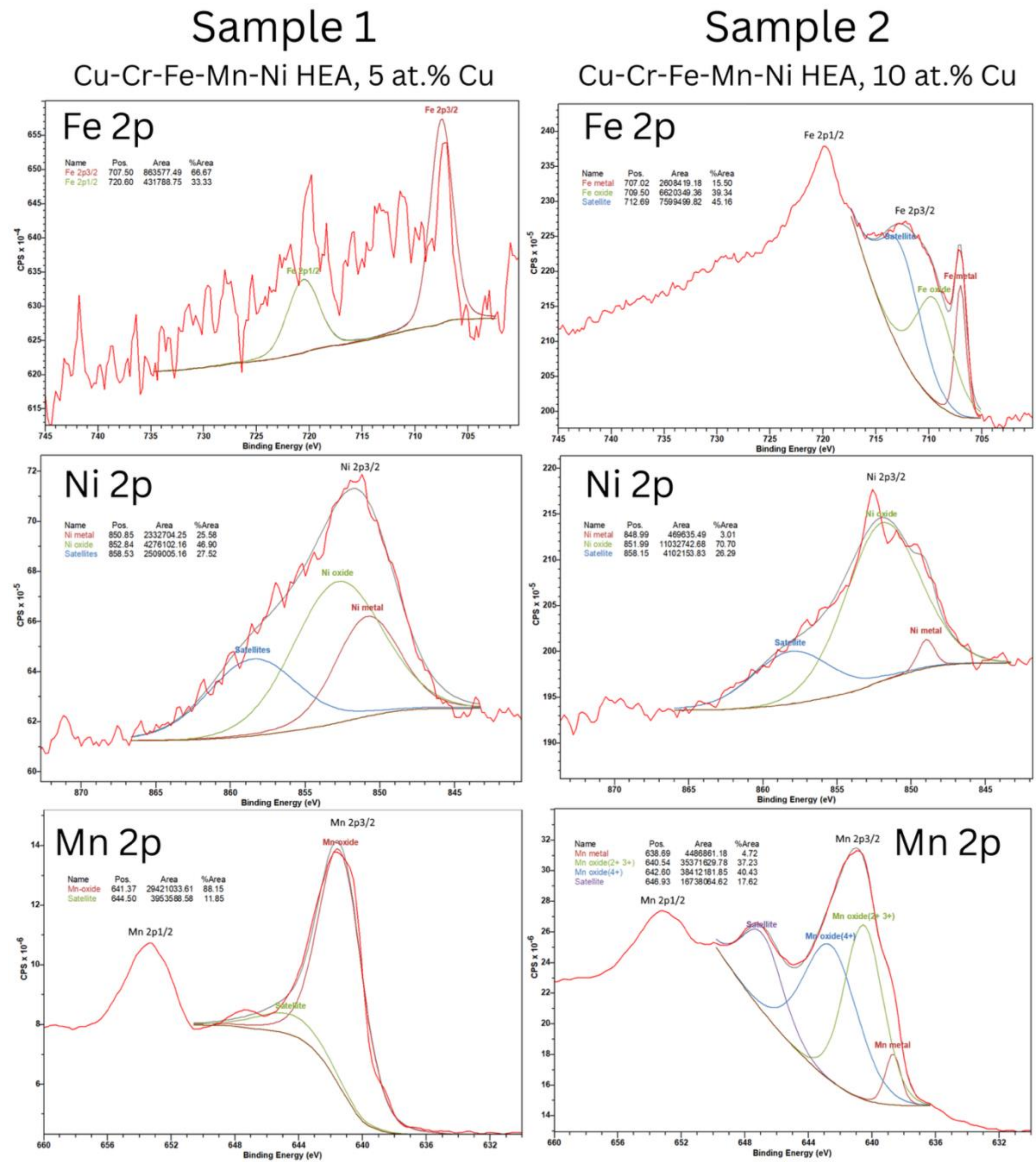

Figure S2. High-resolution XPS spectra of the Fe 2p, Ni 2p, and Mn 2p regions for Cu_x-Cr-Fe-Mn-Ni HEA coatings with 5 and 10 at.% Cu after sputtering, complementing the main XPS analysis shown in Figure 13.

Supplementary note to Fig. 14

Sample 1 (Cu_x-Cr-Fe-Mn-Ni HEA, 5 at.% Cu)

Post-sputtering Fe 2p spectra show low intensity but distinct metallic peaks (~707.0 and ~720.1 eV) with negligible oxide signals, confirming a metallic subsurface. Mn remains fully oxidized, dominated by Mn^{2+}/Mn^{3+} (~641.4 eV) and a ~644.5 eV satellite, indicating a thick,

stable Mn-rich oxide layer. Ni exhibits a mixed state: ~25.6% metallic (~850.85 eV) and ~74.4% oxidized (~852.84 eV with an 858.53 eV shake-up satellite), reflecting a predominantly oxidized surface.

Sample 2 (Cu_x-Cr-Fe-Mn-Ni HEA, 10 at.% Cu)

Fe 2p reveals a mixed state, combining metallic Fe (~707.0 eV) with persistent oxide components (~709.5 eV and ~712.7 eV satellite), indicating subsurface iron oxides. Mn displays heterogeneous oxidation: trace metallic Mn (<5%, ~638.7 eV) alongside dominant Mn^{2+}/Mn^{3+} (~640.5 eV, ~646.9 eV satellite) and a distinct Mn^{4+} shoulder (~642.6 eV). Ni remains heavily oxidized (~97%), characterized by strong Ni^{2+} (~852.0 eV) and satellite (~858.2 eV) signals; the trace metallic fraction (~3%) confirms a robust passive film independent of Cu content.

The Fe 2p, Mn 2p, and Ni 2p binding-energy assignments were made according to literature data [1,2], [3,4], and [5,6], respectively.

Supplementary References

[1] Y. Lou, C. Dai, W. Chang, H. Qian, L. Huang, C. Du, D. Zhang, Microbiologically influenced corrosion of FeCoCrNiMo0.1 high-entropy alloys by marine Pseudomonas aeruginosa, Corros. Sci. 165 (2020). https://doi.org/10.1016/j.corsci.2019.108390.

[2] W. Wang, W. Qi, X. Zhang, X. Yang, L. Xie, D. Li, Y. Xiang, Superior corrosion resistance-dependent laser energy density in (CoCrFeNi)95Nb5 high entropy alloy coating fabricated by laser cladding, Int. J. Miner. Metall. Mater. 28 (2021) 888–897. https://doi.org/10.1007/s12613-020-2238-2.

[3] J. de A. Pereira, J.N. Lacerda, I.F. Coelho, C. de S.C. Nogueira, D.F. Franceschini, E.A. Ponzio, F.B. Mainier, Y. Xing, Tuning the morphology of manganese oxide nanostructures for obtaining both high gravimetric and volumetric capacitance, Mater. Adv. 1 (2020) 2433–2442. https://doi.org/10.1039/D0MA00524J.

[4] H.W. Nesbitt, D. Banerjee, Interpretation of XPS Mn(2p) spectra of Mn oxyhydroxides and constraints on the mechanism of MnO 2 precipitation, Am. Mineral. 83 (1998) 305–315. https://doi.org/10.2138/am-1998-3-414.

[5] P. Yang, Z. Jiang, Y. Shi, W. Zhang, X. Ren, L. Liang, M. Wang, K. Zhu, Construction of high-entropy oxy/hydroxides with dense nanosheet array structures on FeCoNiCrMo electrode for efficient oxygen evolution reaction, Appl. Phys. Lett. 123 (2023). https://doi.org/10.1063/5.0161844.

[6] G.C. Mohanty, C. Chowde Gowda, P. Gakhad, M. Sanjay, A. Singh, K. Biswas, C.S. Tiwary, Effect of Zn/Mn on the supercapacitor behavior of high-entropy FeCoNiCrZn/Mn alloy, Energy Adv. 3 (2024) 2972–2985. https://doi.org/10.1039/D4YA00376D.
